# Far-Field Electrostatic Signatures of Macromolecular 3D Conformation

**DOI:** 10.1021/acs.nanolett.2c02485

**Published:** 2022-09-20

**Authors:** Gunnar Kloes, Timothy J. D. Bennett, Alma Chapet-Batlle, Ali Behjatian, Andrew J. Turberfield, Madhavi Krishnan

**Affiliations:** † Physical and Theoretical Chemistry Laboratory, Department of Chemistry, University of Oxford, South Parks Road, Oxford OX1 3QZ, United Kingdom; ‡ Clarendon Laboratory, Department of Physics, University of Oxford, Parks Road, Oxford OX1 3PU, United Kingdom; ⊥ The Kavli Institute for Nanoscience Discovery, Sherrington Road, Oxford OX1 3QU, United Kingdom

**Keywords:** Electrostatics, electrometry, DNA nanostructures, molecular interactions, 3D conformation

## Abstract

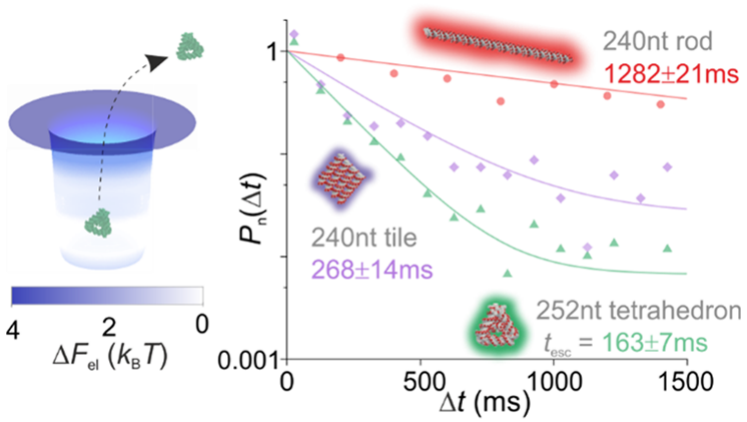

In solution as in vacuum, the electrostatic field distribution in the vicinity of a charged object carries information on its three-dimensional geometry. We report on an experimental study exploring the effect of molecular shape on long-range electrostatic interactions in solution. Working with DNA nanostructures carrying approximately equal amounts of total charge but each in a different three-dimensional conformation, we demonstrate that the geometry of the distribution of charge in a molecule has substantial impact on its electrical interactions. For instance, a tetrahedral structure, which is the most compact distribution of charge we tested, can create a far-field effect that is effectively identical to that of a rod-shaped molecule carrying half the amount of total structural charge. Our experiments demonstrate that escape-time electrometry (ET*e*) furnishes a rapid and facile method to screen and identify 3D conformations of charged biomolecules or molecular complexes in solution.

The structures of materials, phase transitions, chemical reactions, and biological processes fundamentally rely on molecular interactions. Such interactions occur across a range of length scales from short-range bond formation and breakage to long-range Coulombic interactions that stem from the presence of a net molecular electrical charge. The decisive role of three-dimensional molecular shape in short-range interactions and reactive processes is familiar from a wealth of examples provided by stereochemistry and chemical and biochemical selectivity. But molecular shape can also have a strong and measurable impact on the long-range electrostatic interaction.

Consider an isolated electrically charged object in the fluid phase. It is well-known that geometry affects the distribution of electrical potential around the object, in turn influencing the local distribution of counterions.^1−3^ Ion density distributions around a charged object in solution depend significantly on whether the object is a sphere, rod, or a plane, for instance.^4−8^ The influence of object geometry on counterion distribution and dynamics may manifest in a variety of ways in experiment. Some examples of experimental observables include the mobility of a charged object in an electrical field, the osmotic pressure of a suspension of charged particles, ionic strength dependent polyelectrolyte properties such as persistence length as well as in intra- and intermolecular interaction energies and forces.^5,8−12^ Detection of the electrical field or potential distribution at a fixed distance from an isolated test object, using interaction energy measurements, could therefore reveal information not only on the total amount of charge the object carries but also on the details of the 3D distribution of charge.^10^ Importantly, for uniformly charged entities carrying identical amounts of total charge, any differences in interaction energy, measured under identical conditions, should stem solely from the shape of the object.

A significant body of theoretical work discusses the interplay between the geometry of a charged object and its electrostatic properties such as the potential distribution in the surrounding electrolyte and the potential of mean force or interaction energy with another charged entity.^1−7^ These studies generally focus on a parameter called the charge renormalization factor, , that relates the structural charge of the object, *q*_str_, with its effective charge, *q*_eff_. In the molecular context, *q*_str_ denotes the net electrical charge in the molecular structure and is given by the sum of charges carried by ionized structural groups and any bound ions from the electrolyte. The effective charge, *q*_eff_, may depart from the nominal structural value for a variety of reasons. In general charge renormalization theories predict η → 1 for weakly charged objects where the average spacing between charges is much larger than the Bjerrum length, *l*_B_, for charged cylinders or  for charged spheres of radius *R*.^1,5,6^ The Bjerrum length  ≈ 0.7 nm in water is the separation at which the energy of interaction between two elementary charges (*e*) is equal to *k*_B_*T*, where *k*_B_ is the Boltzmann constant and *T* is the absolute temperature. On the other hand, for objects with high charge densities we expect η < 1.^1^ The role of charge renormalization is further accentuated under conditions of weak electrostatic screening, with η generally decreasing as the salt concentration decreases.^5,6,8^ Importantly, since η depends on the 3D distribution of charge in an object, *q*_eff_ depends strongly on the shape of the object.^4^

Although the renormalization factor has most often been discussed in the context of the effective charge of an isolated charged object, we have recently found that the value calculated for an isolated object turns out to be quantitatively very similar to the effective charge that determines the electrical free energy in a molecular interaction (between the object of interest and another charged entity).^5,6,8,13,14^ Our previous experimental work on a range of different biomolecules has further demonstrated that measurements of effective charge, as inferred from the interaction of a charged molecule with a like-charged flat surface in solution for example, correspond remarkably well to *q*_eff_ values calculated using Poisson–Boltzmann (PB) interaction free energies.^8,10^ Further, it is worth noting that our interaction based definition of effective charge is the same as that used in Kjellander’s “dressed ion” theory.^13,14^

In this study we use DNA nanostructures to examine the impact of 3D macromolecular conformation on long-range molecular electrostatic interactions and, conversely, to explore the possibility of using high-precision electrostatics measurements to shed light on 3D macromolecular conformation and structure.^15^ We measure electrostatic interaction energies and corresponding effective charge values for various DNA constructs confined between two parallel charged plates at a separation 2*h*. We work in the regime given by κ*h* ≈ 4–8, i.e., where the typical separation, *h*, between molecule and probe is greater than the Debye length, κ^–1^. Here κ^–1^ = (8π*l*_B_*N*_A_*I*)^−1/2^ is a length scale which characterizes the rate of exponential decay of the electrical potential with distance from the surface of a charged object, where *N*_A_ is Avogadro’s number and *I* is the ionic strength of the electrolyte (κ^–1^ = 4–9 nm in this study).

Here, we consider DNA nanostructures designed to carry effectively the same total structural charge, *q*_str_, but of four different molecular shapes representing conformations of varying “compactness”, as reflected in their respective radii of gyration, *R*_g_, the root-mean-square distance of all the atoms in the molecule from its center of geometry (Table S5). Beginning with a 120 bp approximately cylindrical double-helix (*q*_str_ = −238 *e*; *R*_g_ = 12 nm), we consider progressively more compact molecular 3D conformations given by a 2-helix bundle (*q*_str_ = −237 *e*; *R*_g_ = 6 nm), a square-tile (*q*_str_ = −237 *e*; *R*_g_ = 4 nm), and finally a DNA tetrahedron (*q*_str_ = −250 *e*; *R*_g_= 3.75 nm)^16^ (Supporting Information section 1). For reference, we also examine a 60 bp DNA cylindrical double-helix bearing half the amount of structural charge carried by the other constructs (*q*_str_ = −120 *e*; *R*_g_ = 6 nm).

## Measuring the Effective Charge of DNA Nanostructures Using Escape-Time Electrometry (ET*e*)

**Figure 1:**
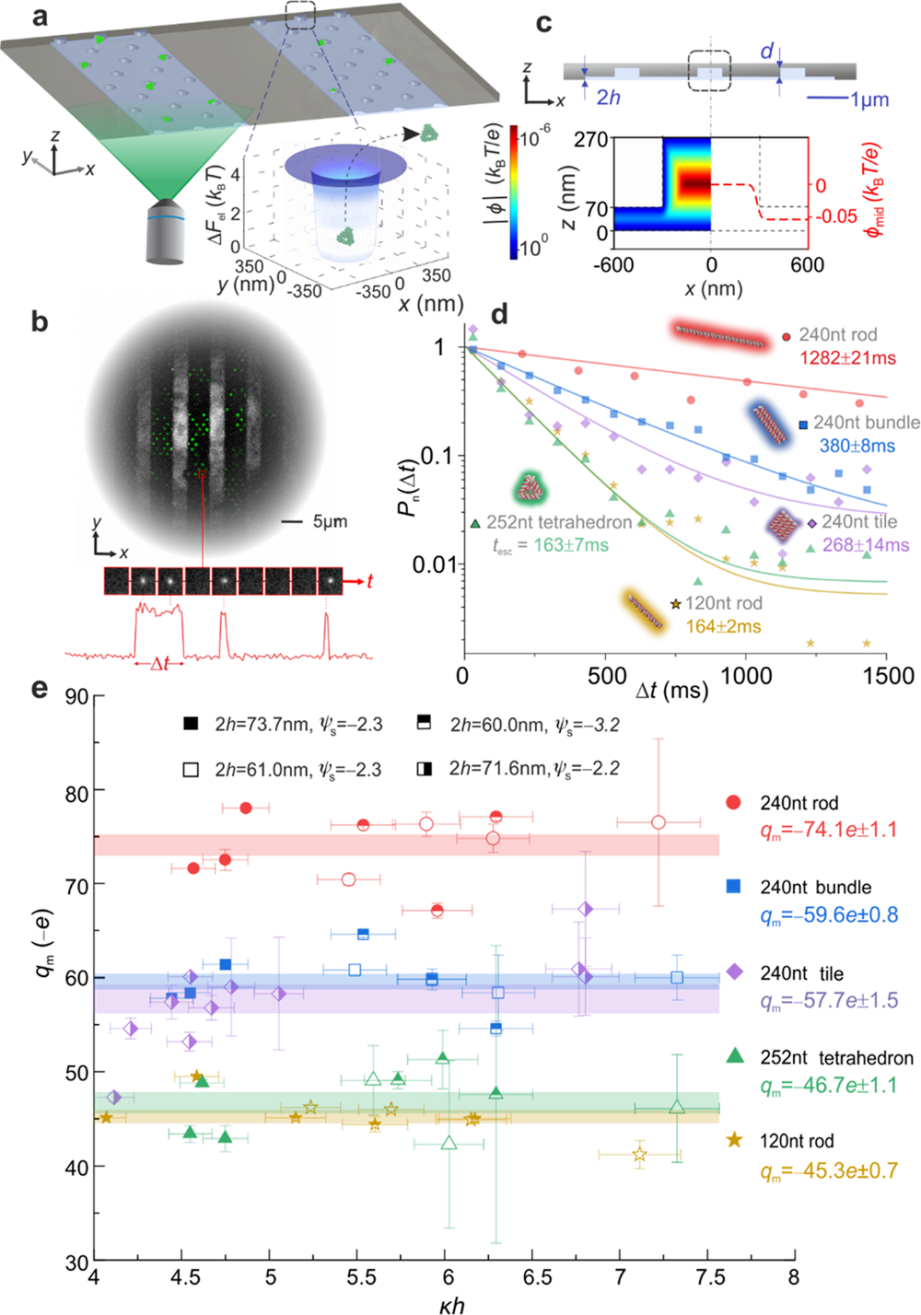
Measurement of effective charge of DNA nanostructures using ET*e*. (a) Graphic representation of an ET*e* device consisting of multiple parallel fluidic slits housing arrays of electrostatic fluidic traps, imaged using wide-field fluorescence microscopy. Calculated representative electrostatic energy landscape (Δ*F*_el_) for a single trap (inset). (b) Maximum intensity projection of the fluorescence signal recorded over a time period of 20s in a typical ET*e* experiment (top). Temporal evolution of the fluorescence signal in a representative single trap displaying a series of images and the corresponding intensity trace (bottom). (c) Graphical representation of the cross-section of a fluidic slit of height 2*h* ≈ 70 nm and pocket depth *d* ≈ 200 nm (top). Below: Calculated electrostatic potential distribution, ϕ, presented on logarithmic scale for a salt concentration *c* = 1.5 mM and *h* = 35 nm (left half-space) and line plot of midplane potential value, ϕ_mid_, in the vicinity of a single pocket (right half-space), displaying a value ϕ_m_ ≈ −0.05*k*_B_*T*/*e* in the slit region, corresponding to an effective surface potential of ϕ_s_ = −2.3*k*_B_*T*/*e*. (d) Probability density distributions of residence times, obtained from 10 to 20 min of imaging, are fitted to the expression *P*(Δ*t*) ∝ exp(−Δ*t*/*t*_esc_) to obtain an average escape time, *t*_esc_, for each nanostructure species. *P*(Δ*t*) distributions are presented such that the maximum value of the fitted curve is set to 1 in order to foster visual comparison of *t*_esc_ for all species measured. *t*_esc_ for species measured under identical conditions may be compared directly. A factor 8 difference in *t*_esc_ values between the tetrahedron (*t*_esc_ = 163 ± 7 ms) and the 120 bp rod (*t*_esc_ = 1282 ± 21 ms) implies a substantially smaller Δ*F*_el_ value for the former. Note that in general we do not include in the fitting process 1–2 data points at the shortest measured lag times, Δ*t*. This is because escape events recorded at the shortest durations can contain contributions from transiently trapped, weakly charged molecular species such as free dye, degraded material, or free DNA strands in the background solution. All measurements were performed in devices with slits of depth 2*h* = 71.6 nm and surface potential value ϕ_s_ = −2.3*k*_B_*T*/*e*, under similar electrolyte conditions (κ*h* ≈ 4.5). Measurements on the square-tile, however, were performed in a similar device but with 2*h* = 73.7 nm and ϕ_s_ = −2.2*k*_B_*T*/*e*, yielding a *t*_esc_ value that is not directly quantitatively comparable with values for the other species shown. (e) *q*_m_ values inferred for each individual measurement for all species are constant within measurement uncertainty for a given nanostructure species, over a range of salt concentrations (*c* = 1.1–5.5 mM) as well as across different measurement devices where slit heights ranged from 2*h* ≈ 60.3 to 73.7 nm and dimensionless surface potentials ranged from ψ_s_ =  = −3.2 to −2.2. Also noted are *q*_m_ values averaged across the range of κ*h* probed.

We performed electrostatic interaction energy measurements on the DNA nanostructure species, labeled with one ATTO 532 fluorophore per nanostructure, using our recently developed escape time electrometry (ET*e*) technique^10,17−19^ (Figure 1). ET*e* offers high precision measurements of electrostatic interaction energies and therefore of molecular effective charge in the fluid phase. Briefly, we used wide-field fluorescence microscopy to image the escape dynamics of single molecules, loaded into a system of multiple parallel slits each containing lattices of traps as shown in Figure 1. We first extracted precise measurements of the average molecular time to escape, *t*_esc_, (Figure 1b,d) and then converted *t*_esc_ to electrostatic interaction free energies, Δ*F*_el_. Next, we deduced measured effective charge values, *q*_m_, as in previous work^10,17−19^ (Figure 1a). A full description of the details of the ET*e* measurement is provided in the Supporting Information section 2.

In our work, the relationship between Δ*F*_el_ and molecular effective charge, *q*_eff_, is given by Δ*F*_el_ = *q*_eff_Δϕ_mid_, where ϕ_mid_ is the electrical potential at the midplane of a parallel-plate system, and Δ denotes the difference between two states of the molecule where it is outside and inside the trap region (also referred to as the “slit” and “pocket” states), as shown in Figure S8a of the Supporting Information. This can be simplified to Δ*F*_el_ = *q*_eff_ϕ_m_, where ϕ_m_ ≈ Δϕ_mid_ ≈ 2ϕ_s_ exp(−κ*h*) is the electrical potential in the nanoslit region of height 2*h*, since ϕ_mid_ in the pocket region is zero by design (Figure 1c). Here, ϕ_s_ is the effective surface electrical potential at the silica walls of the slit, and κ*h* is a dimensionless system size or a dimensionless separation distance between a molecule and a slit surface. It follows that
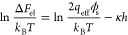
1The two unknowns in eq 1 are *q*_eff_, which describes the molecule of interest, and ϕ_s_ which describes the measurement device. In order to determine the value of ϕ_s_ in a given experiment, we first performed a calibration measurement using a well-characterized molecular species of known effective charge under experimental consitions, namely 60bp double-stranded DNA fragment carrying two fluorophore labels characterized by *q*_eff_ = −46.2 *e*.^10,17,19^ Values for ϕ_s_ obtained in four different measurement devices are noted in Figure 1e and are consistent with previously measured values.^10,17−19^ Measurements of *t*_esc_ under various conditions of monovalent salt concentration (*c* = 1.1–5.5 mM; κ^–1^ = 4–9 nm) and slit height are converted to electrostatic free energies, Δ*F*_el_, which are plotted against κ*h*. A fit to eq 1 in conjunction with the value for ϕ_s_ yields measured values of *q*_eff_ for each molecular species, as discussed in detail in Supporting Information section 2.5. Measured effective charge values, denoted as *q*_m_ are displayed in Figure 1e, displaying constant behavior over the range of κ*h* probed in experiment. Although *q*_eff_ is expected to depend on salt concentration, we do not expect significant variation over the narrow range of *c* probed in these experiments (Supporting Information section 3.3).^5,6,8^ Finally, we work with a measurement uncertainty of ≈2–7% on *t*_esc_ and on the molecular effective charge (Supporting Information section 2.6), which is sufficient to delineate broad trends arising from molecular geometry.

## Calculated Effective Charge Values for DNA Nanostructures

**Figure 2:**
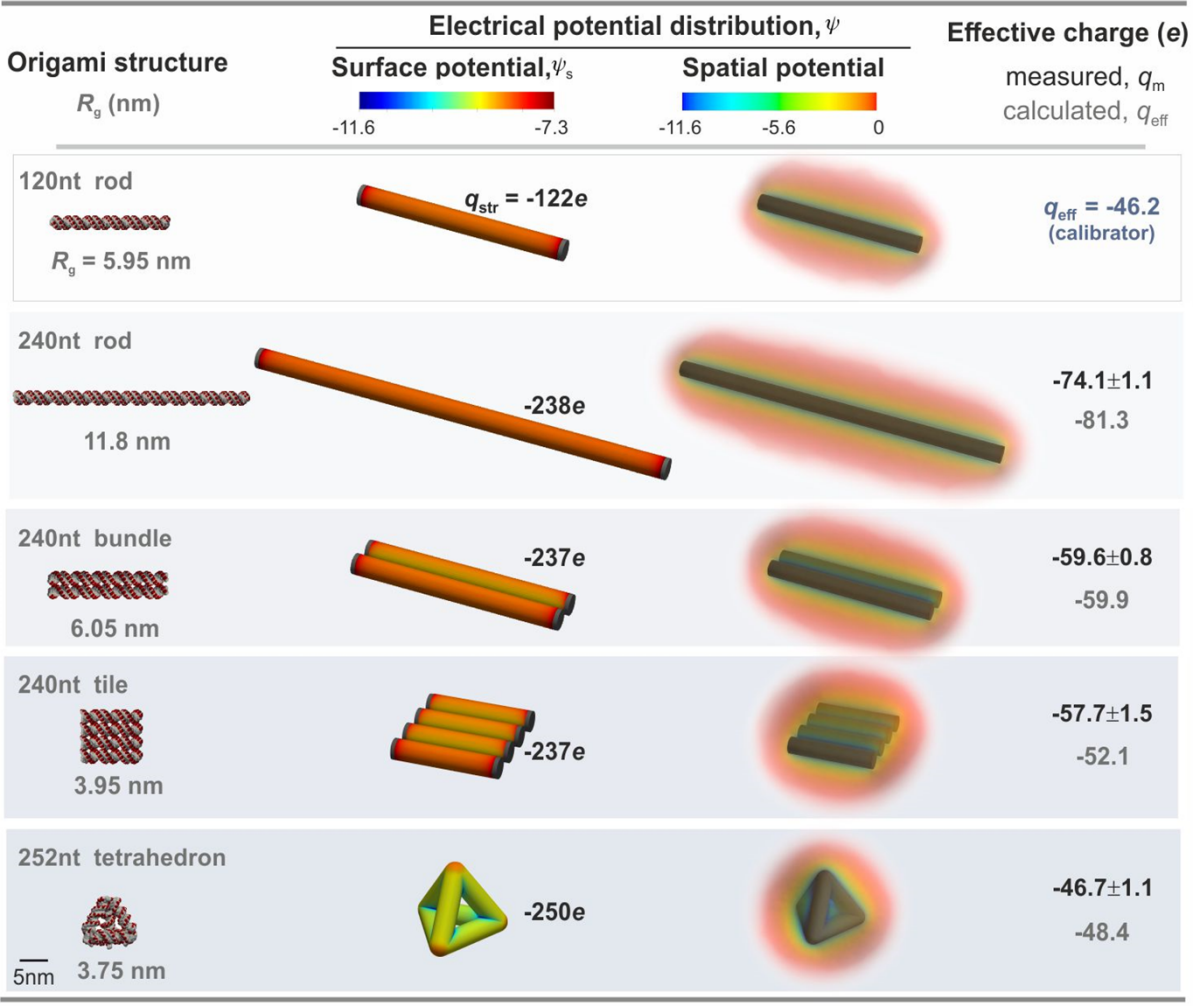
Electrostatic modeling and effective charge measurements for DNA nanostructures. Columns 1 and 2 list values for the radius of gyration, *R*_g_ (computed for all atoms in the molecular structures), DNA nanostructure size in nucleotides (nt), and total structural charge, *q*_str_, including the contribution of the fluorescent dye. Electrostatic modeling of the nanostructure was performed by solving the PB equation for coarse-grained cylinder-based models of the nanostructures immersed in an electrolyte containing 1.5 mM NaCl (described further in Supporting Information section 3). Distributions of dimensionless surface and spatial electrical potential, ψ_s_ (column 2) and ψ (column 3), respectively, are calculated for each nanostructure model. Spatial distributions of electrical potential, ψ, correspond to the local electrolyte volume surrounding the nanostructures. ψ_s_ and ψ are presented on an identical scale of electrical potential for all nanostructures and emphasize the sensitivity of the spatial potential distribution around the object to 3D molecular conformation and “compactness”. Column 4 compares calculated effective charge values, *q*_eff_ (bottom), with measured effective charge values, *q*_m_ (top), that are averages over the series of ET*e* measurements presented in Figure 1e.

**Figure 3:**
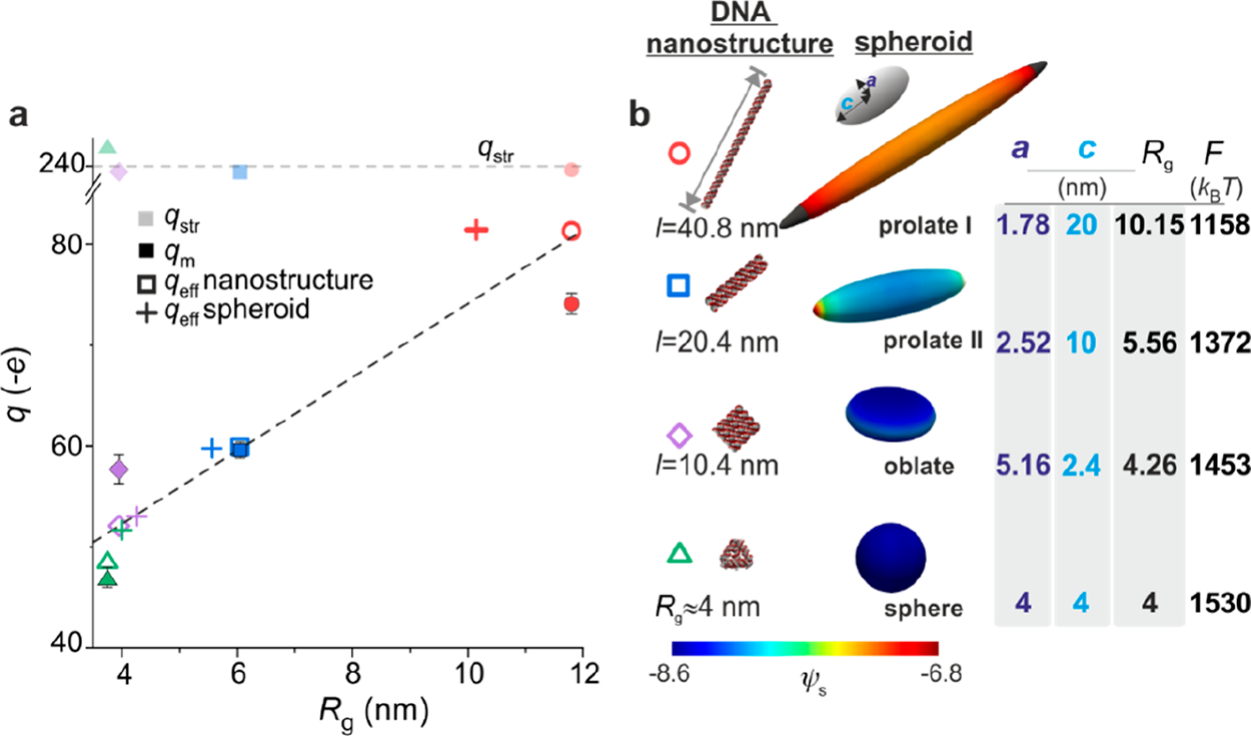
Interpreting the 3D conformation dependence of nanostructure effective charge. (a) Measured *q*_m_ (dark solid symbols) and calculated *q*_eff_ (open symbols) effective charge values for each DNA nanostructure species increase in magnitude with increasing radius of gyration *R*_g_. Dashed lines are guides for the eye. (b) The trend observed for DNA nanostructures is captured by charged hollow spheroids of fixed total volume, , where *R* = 4 nm and *q*_str_ = −240 *e*. *q*_m_ and *q*_eff_ values for the nanostructures are very similar to *q*_eff_ values for spheroidal surface charge distributions of similar *R*_g_ (“plus” symbols in (a)). We use a sphere of radius *R* = 4 nm to model the DNA tetrahedron, prolate spheroids I and II to mimic the rod and the bundle respectively, and an oblate spheroid to model the square-tile. In each case we set the length of the longer semiaxis to correspond to half the longest dimension of the corresponding nanostructure. The table lists semiaxis lengths, *a* and *c*, for the spheroids, as well as electrical free energies, *F*, calculated using eq S14 in Supporting Information for each spheroid in free solution (right). *F* displays an increase with increasing sphericity, whereas *q*_eff_ decreases with increasing sphericity. Surface electrical potential, ψ_s_, distributions display a systematic reduction in magnitude with increasing *R*_g_ of the spheroid, correlating with an increase in magnitude of *q*_eff_. Note that because all the ionized groups reside on the molecular surface, we use *R*_g_ values for hollow rather than solid geometries for the spheroids as well as for the coarse-grained molecular models in Figure 2.

Despite the fact that all our test DNA nanostructures carry nearly identical amounts of the structural charge, we note a gradual reduction in the magnitude of *q*_m_ of nearly a factor of 2 from the most extended nanostructure (with the largest value of *R*_g_), the 120 basepair double-helix, to the most compact, the tetrahedron. We further calculated theoretically expected values of the effective charge of the DNA nanostructures using an approach described previously^8^ and outlined in detail in the Supporting Information section 3. Individual nanostructures were modeled as an assembly of smooth charged cylinders each representing a segment of double helical DNA. The cylinders have a radius *r*_cyl_ = 1.2 nm, a length corresponding to a rise per base pair value of 0.34 nm, and carry a uniform charge density σ = *q*_str_/*A*, where *A* is the total surface area of the structure.^31^ Distributions of dimensionless electrical potential  on the surfaces of the nanostructures (ψ_s_) as well as in the surrounding electrolyte in the vicinity of the nanostructure (ψ) are displayed in Figure 2. We note remarkable agreement in general between the experimental *q*_m_ values and the calculated *q*_eff_ values in each case (Figures 2 and 3). We further found that a tetrahedral arrangement of a total electrical charge of *q*_str_ = −250 *e* (*R*_g_ ≅ 4 nm) is in fact nearly indistinguishable in the present measurement from a linear arrangement of half that amount of charge, as encountered, for example, in a fragment of 60 bp DNA (*q*_str_ = −122 *e*; *R*_g_ ≅ 6 nm). We did however note a 10% discrepancy between the measured and calculated effective charge values for the 120 bp double-helix (Figure 2). This disparity may arise from the fact that whereas we model the double-helix as a rigid cylindrical rod, the 120 bp fragment of DNA in our experiments may be less extended as it is assembled from four oligonucleotides (see Supporting Information section 1), possibly resulting in a structure that is more flexible than a canonical two-strand double-helix.

In order to illustrate the ability of a measurement of *q*_eff_ to provide information on molecular 3D geometry, we mapped the nanostructure electrostatics problem onto that of charge-carrying spheroids of fixed total volume *V* and total charge *q*_str_ = −240 *e*. We set the volume of each spheroid  to be equal to that of a spherical shell of radius equal to the radius of gyration of the DNA tetrahedron (*R*_g_ ≈ 4 nm), our most conformationally compact nanostructure. We then varied the semiaxis lengths *a* and *c* in order to capture the range of nanostructure shapes in the study. We then calculated effective charge values for hollow, uniformly charged spheroids representing each nanostructure. As reflected in the PB equation (Supporting Information S3), charge renormalization is a manifestation of nonlinearity in the underlying electrostatics. A low-density charge distribution generally places the electrostatics problem in the linear regime, characterized by |ψ| < 1, where *q*_eff_ → *q*_str_. A more compact or dense charge distribution would generally imply a large value of |ψ|, particularly in the vicinity of the object, and we expect *q*_eff_ ≪ *q*_str_ as a consequence^5,8^ (Figures 2 and 3). In line with these expectations, we observe that more extended nanostructures and spheroids display lower magnitudes of electrical potential, both at the molecular surface (ψ_s_) and in the electrolyte (ψ), and are associated with larger magnitudes of effective charge (Figure 3). Comparing calculations of effective charge with the measured values for DNA nanostructures as a function of *R*_g_ reveals that the overall trend in measured effective charge, *q*_m_, is captured well by *q*_eff_ values calculated for the equivalent spheroids (Figure 3). This analysis recovers a feature that is in line with theorems on the geometry dependence of the electrostatic energy and capacity of isoperimetric, charged, conducting solids immersed in a dielectric medium, which have been previously applied to the study of macromolecular topological properties such as knots in polyelectrolytes.^20−24^ Our analysis reveals that the most compact charged objects (the sphere and the DNA tetrahedron) have the highest electrical free energy and also the lowest effective charge which may be viewed as analogous to the electrostatic capacity (Figure 3). Our results provide further confirmation that electrometry measurements, in combination with information on a charged molecular species such as a molecular weight estimate, can yield coarse-grained structural information on the 3D morphology of a charged molecule.

**Figure 4:**
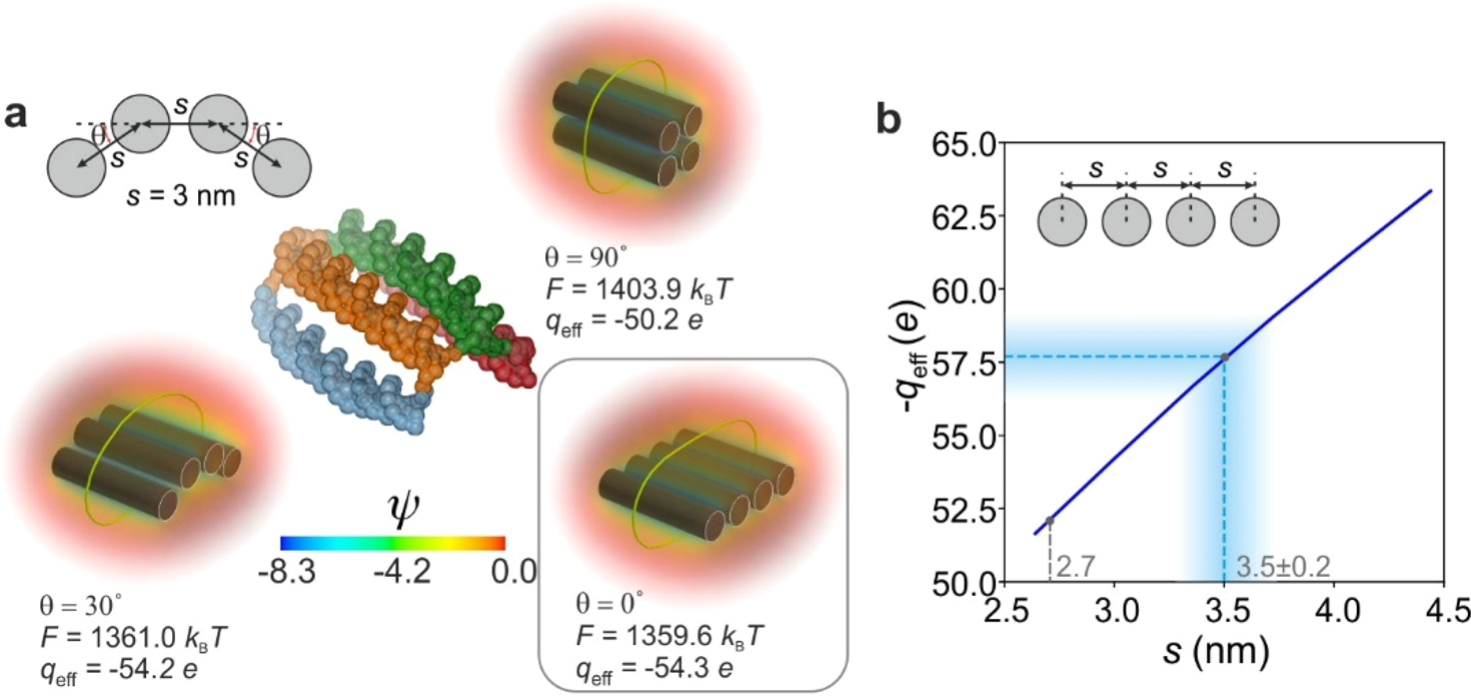
Interhelix spacing in the square-tile nanostructure inferred from its measured effective charge. (a) Calculated electrostatic free energies, *F*, in free solution (calculated using Supporting Information eq S14) and associated effective charge values, *q*_eff_, are shown for three different tile geometries, each characterized by a spacing *s* = 3 nm and the quoted angle θ for nonplanar structures. The planar structure with θ = 0° corresponds to the state with the minimum electrostatic free energy. Electrical potential distributions, ψ, depict the electrolyte volume surrounding each nanostructure. (b) Plot of calculated *q*_eff_ vs *s* for a planar square-tile (θ = 0°). Our measured effective charge, *q*_m_ = −57.7 ± 1.5 *e*, points to a relatively expanded square-tile structure with an interhelical spacing of *s* = 3.5 ± 0.2 nm.

Finally, we focus on the 3D conformation of the square-tile. The measurement indicates a value of *q*_m_ = −57.7 ± 1.5 *e*, which is significantly larger in magnitude than the nominal value of *q*_eff_ = −52.1 *e* calculated for a planar square-tile structure with an interhelix spacing *s* = 2.7 nm as reported in X-ray scattering measurements.^25,26^ We calculated electrostatic free energies in free solution, *F*, and *q*_eff_ values for various conformations of the square-tile, including a “bent” structure representing the average conformation from 2000 time-steps of a molecular simulation using oxDNA^27,28^ (Figure 4a). Under our measurement conditions the planar conformation is suggested to be the thermodynamically favored state as it has the lowest calculated value of *F* (Figure 4a). In general, we expect greater conformational compactness to yield larger electrostatic free energies, corresponding to smaller free energy differences in the trap and therefore smaller magnitudes of *q*_eff_. Therefore, an experimental measurement of *q*_m_ larger in magnitude than the *q*_eff_ value of a reference conformational state would *ipso facto* point to a more expanded molecular conformation. Varying the interhelix separation, *s*, of the planar conformation of the tile in calculations, we find agreement between our measured *q*_m_ value and the calculated *q*_eff_ value for a structure with *s* = 3.5 ± 0.2 nm, about 30% larger than the literature value (Figure 4b). This may be explained by the fact that our measurements are performed in solutions containing ∼1–6 mM NaCl where weaker electrostatic screening could be expected to favor a more expanded molecular state. In general, our measurements of the renormalization factor, η, are in good agreement both with our calculated values and with prior theoretical estimates for charged rods and disks (Supporting Information section 3.4).^29,30^ Thus, our study further suggests that beyond detecting coarse-grained morphological features of molecular 3D conformation (e.g., elongated/unfolded or compact/globular), precise measurements of electrostatic energies may further shed light on finer-grained structural attributes such as periodicities and spacings in ordered molecular charge distributions.^31^

Our interaction-based definition of molecular effective electrical charge implies that the free energy of interaction between a molecule and a charged entity in solution may be written as *F*(**r**) = *q*_eff_ϕ(**r**), where ϕ(**r**) is the electrical potential created by the charged entity at the location of the molecule, **r**.^8^ This definition of interaction free energy is the same as the potential of mean force between a charged object generating a potential distribution ϕ(**r**) in the electrolyte and a macroion of effective charge *q*_eff_ located at **r**.^13,14^ Since the corresponding force can be written as *f*(**r**) = −*q*_eff_∇ϕ(**r**), measurements of effective charge can yield a measure of the expected force in the interaction of a molecular species of interest with another charged object in solution. Although our measurements concern the interaction of a molecular scale entity and a like-charged plate, the theoretical basis of the underlying interaction permits us to extrapolate the observed shape-dependence to the interaction between charged molecules in solution.^13,14^ The short-range consequences of sequence specificity and precise local structural detail in nucleic acid–protein and protein–protein interactions are well established.^32−34^ We have demonstrated that folding, compaction, or conformational collapse of a charged object can reduce the magnitude of the electrostatic energy or interparticle force in an interaction, on account of the effect of *molecular 3D shape alone* on the long-range electrostatics. Macromolecular folding or compaction would normally entail additional energetic effects including contributions from charge regulation or the shifting of acid–base equilibria of ionizable groups and the formation of and exclusion of water from a low dielectric molecular interior,^35−37^ all of which might be expected to reduce the structural charge of the molecule by lowering the extent of ionization of its chargeable sites.^38^ However, our study utilizes molecular species with highly acidic ionizable groups that retain a large degree of exposure to the solvent, enabling us to probe the impact of molecular 3D conformation alone on long-range electrostatic interactions. Our observations indicate that in an interaction with a charged entity, oligonucleotides with the same number of bases but in different states of compaction experience electrostatic interactions that are significantly different, suggesting a functional role of secondary and tertiary structure in regulating longer-range intermolecular interactions. In other words, molecular shape matters even at long range. We point out that our observations pertain to a Poisson–Boltzmann mean field description of the electrostatic part of an interaction alone, which acts in concert with a number of additional mechanisms, e.g., dispersion, solvation, and fluctuation forces, depending on experimental conditions.^37−41^ Finally, our ability to detect differences in the 3D distribution of charge in a molecule is in line with expectations from a rigorous theoretical treatment of multipolar charge distributions in electrolytes.^2,3,14^

In conclusion, the ability to experimentally detect differences in 3D conformation has immediate and obvious implications for the rapid, solution-phase characterization of biomolecular structure and conformation, particularly for strongly charged nucleic acids. For example, we envision the ability to assess the conformations of complex RNA molecules, where structural studies lag far behind those for proteins.^42^ The experiments performed in this study were designed to furnish a proof of the principle that precise measurement of electrostatic interactions can yield information on 3D macromolecular conformation in solution. Although ensemble-averaging in data analysis means that measurements reflect the statistically dominant conformation, we expect that in future, analyzing escape time data on the basis of migration trajectories of individual molecules could provide a view of the “conformation spectrum” of a mixture of chemically similar yet structurally and/or conformationally distinct molecular states.

## Supporting Information Available

Supporting Information (PDF): . The Supporting Information is available free of charge at https://pubs.acs.org/doi/10.1021/acs.nanolett.2c02485.

(1) DNA nanostructure synthesis and characterization; (2) escape-time electrometry (ET*e*) to measure the effective charge, *q*_m_, of DNA nanostructures; (3) procedure to calculate values of effective charge, *q*_eff_, for DNA nanostructures (PDF)

## Notes

Raw data and analysis scripts are available upon request.
