# Supplementary material for: Far-Field Electrostatic Signatures of Macromolecular 3D Conformation
Source: Nano Lett. 2022 Sep 20;22(19):7834–40. doi: 10.1021/acs.nanolett.2c02485 (PMC9562458; doi:10.1021/acs.nanolett.2c02485)
Supplement: Supplementary file 1 — nl2c02485_si_002.pdf [file nl2c02485_si_002.pdf]

Supporting Information for

Far-field electrostatic signatures of macromolecular 3D conformation

Gunnar Kloes†1, Timothy J. D. Bennett†1, Alma Chapet-Batlle[2], Ali Behjatian[1], Andrew J. Turberfield[2] & Madhavi Krishnan[1]*

[1]Physical and Theoretical Chemistry Laboratory, Department of Chemistry, University of Oxford, South Parks Road, Oxford OX1 3QZ, United Kingdom

[2]Clarendon Laboratory, Department of Physics, University of Oxford, Parks Road, Oxford OX1 3PU, United Kingdom

*Corresponding author. Email: madhavi.krishnan@chem.ox.ac.uk

†these authors contributed equally to the work

Table of contents:

1. **DNA Nanostructure synthesis and characterisation……………………………….2**

   Figures S1-5

   Tables S1-5

2. **Escape-time electrometry (ET*e*) to measure the effective charge, q_{m}, of DNA nanostructures……………………………………………………………………15**

   Figures S6,7

3. **Procedure to calculate values of effective charge, q_{eff}, for DNA nanostructures……………………………………………………………………30**

   Figures S8,9

Supporting References………………………………………………………………...37

1. DNA Nanostructure synthesis and characterization

1.1: Nanostructure characterization and general procedure for synthesis

Sequences. Oligonucleotide sequences are recorded in Tables S1-4.

Oligonucleotide synthesis. DNA oligonucleotides were ordered from Integrated DNA Technologies, Inc (IDT), unpurified except for those with the 5'Atto-532 fluorescent label (HPLC purified). Strands were resuspended at a concentration of 100μM in TE Buffer (10mM Tris + 1mM EDTA).

Assembly. Annealing protocols for each nanostructure are specified separately below.

Gel characterization. Nanostructures were characterized by polyacrylamide gel electrophoresis (PAGE) using 7.5% polyacrylamide gels (29:1 acrylamide : bisacrylamide) run in 1× TAE buffer (40mM Tris, 20mM acetic acid, 1mM EDTA) at 180 V for 35 min. Gels presented in Figures S1-5 were imaged in the Atto532 fluorescence channel (532nm laser and 570nm bandpass filter) with an Amersham Typhoon Gel Imaging System (GE Healthcare).

Gel purification. Gels (run as above) were imaged in the Atto532 fluorescence channel (532nm laser and 570nm bandpass filter). The band corresponding to a fully-assembled nanostructure was cut out and nanostructures eluted by adding 200 μL TE Buffer with 5 mM $MgCl_2$ (tetrahedron, two-helix bundle, 120bp DNA helix) or 12.5 mM $MgCl_2$ (square tile), freezing and thawing twice and collecting the supernatant. Tetrahedron and square tile samples were further concentrated by ethanol precipitation[1] and resuspended in TE Buffer alone (square tile) or in TE Buffer with 5 mM MgCl2 (tetrahedron).

Quantification. Purified sample concentrations were estimated on the basis of the intensity of emission from the Atto532 fluorophore. Fluorescence intensities were recorded using a CLARIOstar Plus (BMG Labtech) Microplate reader (excitation: 520nm, emission: 570nm). A calibration curve was obtained using the 5'Atto-532 labeled strand T_s1 at known

concentrations ranging from 100 pM to 100 nM. Measurements were performed in triplicate and averaged.

1.2: Tetrahedron synthesis

Design. The tetrahedron was synthesized as described in Goodman *et al.* [2] with the addition of an Atto532 fluorescent label on strand T_s2 (Figure S1a).

Tetrahedron Assembly. Labeled strand (T_s2) at 50nM and unlabeled strands (T_s1, T_s3, T_s4) at 75 nM were combined in TE Buffer with 5 mM MgCl. The mixture was heated to 95°C, held for one minute, then cooled at 2°C per minute to 22°C.

1.3: Square tile synthesis

Design. The tile comprises four DNA double-helices, each of length of 30 bp, bound to neighboring helices through crossovers at the helix ends. Nicks (Figure S2a,b, helix 1, 2 and 3) were placed asymmetrically, dividing a helix into 20bp and 10bp domains, in order to reduce the potential for aggregation during assembly.

Assembly. Labeled strand (Sq_s1) at 50 nM and unlabeled strands (Sq_s2, Sq_s3, Sq_s4, Sq_s5) at 75 nM were combined in TE Buffer with 12.5 mM MgCl. The mixture was heated to 95°C, held for one minute, then cooled at 1°C per minute to 22°C.

1.4: Tetrahedron synthesis

Design. The two-helix bundle is a double crossover tile[3] comprising two 60-nucleotide DNA helices bound together by two crossovers. It is assembled from five strands of DNA, two that run the full length of the helices and three that bind these two strands together (Figure S3a,b). There is a single nick on one of the helices where the 5' and 3' ends of the central linking strand meet. The spacing between the central crossovers was chosen to be 21 bp, corresponding to two turns of the DNA double-helix.

Assembly. Labeled strand (S2) at 200 nM and unlabeled strands (Figure S3, B_s1, B_s2, B_s3) at 300nM were combined in TE Buffer with 12.5 mM MgCl. The mixture was heated to 95°C, held for one minute, then cooled at 1°C per minute to 22°C.

1.5: 120bp DNA helix synthesis

Design. The 120bp helix is assembled from four strands of DNA to circumvent the requirement to synthesize an Atto532-labeled oligonucleotide of length 120 nucleotides (Figure S4a). Strands S1 and S2 from the 2-helix bundle are re-used in this structure.

Assembly. Labeled strand (S1) at 200nM and unlabeled strands (S2, Ds_s1, Ds_s2) at 300 nM were combined in TE Buffer with 12.5 mM MgCl. The mixture was heated to 95°C, held for one minute, then cooled at 1°C per minute to 22°C.

1.6: Determining the structural charge of DNA nanostructures

The structural charge, q_{str}, is determined for each of the DNA nanostructures as follows. Beyond summing the number of bases, n_{b} (each associated with a charge of -1*e* due to a phosphate ester group), we also consider the number of strands, N_{s}, in each structure, and whether their 5' termini are phosphorylated or not. In the case of all the nanostructures, ($N_{\mathrm{s}} -$ 1) strands had hydroxylated 5' termini, while the dye-carrying "N_{s}th strand" did carry a 5' phosphorylation to which the dye is chemically coupled. The 60bp double-stranded calibrator molecule had both strands phosphorylated at the 5' end. We also add a contribution of -1*e* to the total structural charge for each Atto-532 dye attached to the DNA nanostructure, as quoted on the manufacturer's website (https://www.atto-tec.com/support/datatable/) and independently verified in previous ET*e* measurements[4]. Table S5 summarizes the charge calculation.

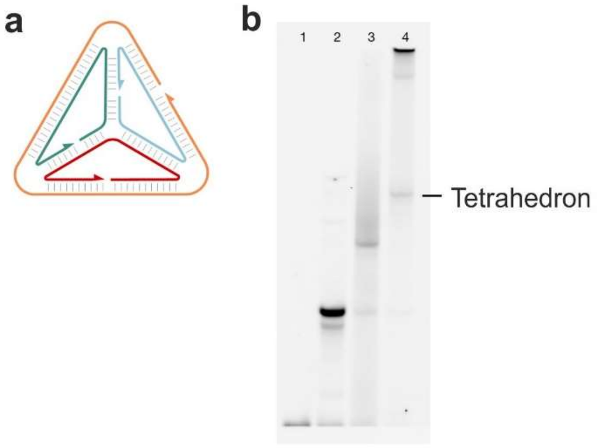

Figure S1. Design & assembly of the tetrahedron. (**a**) Tetrahedron (Figure adapted from Goodman *et al.* [2]) (**b**) Formation gel: each lane contains the unpurified products of assembly of one more strand than the previous lane. Lane 1: T_s1; Lane 2: T_s1 + T_s2; Lane 3: T_s1 + T_s2 + T_s3; Lane 4: T_s1 + T_s2 + T_s3 + T_s4.

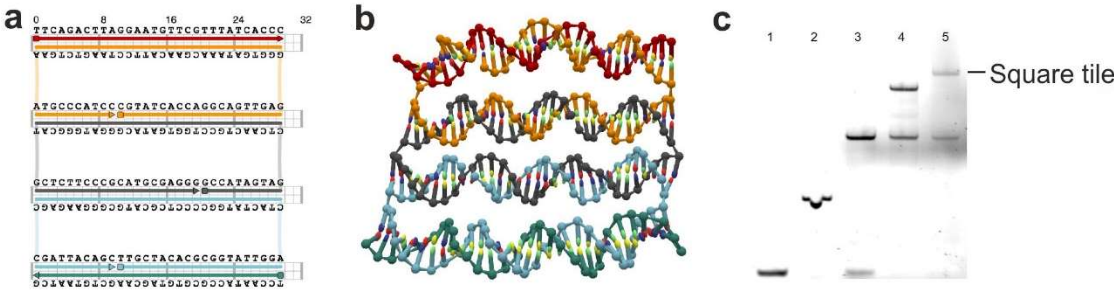

Figure S2. Design & assembly of the square tile. (**a**) scadnano[5] design of the square tile. (**b**) OxDNA[6-8] representation of the square tile (**c**) Formation gel: each lane contains the unpurified products of assembly of one more strand than the previous lane. Lane 1: Sq_s1; Lane 2: Sq_s1 + Sq_s4; Lane 3: Sq_s1 + Sq_s4 + Sq_s2; Lane 4: Sq_s1 + Sq_s4 + Sq_s2 + Sq_s5; Lane 5: Sq_s1 + Sq_s4 + Sq_s2 + Sq_s5 + Sq_s3.

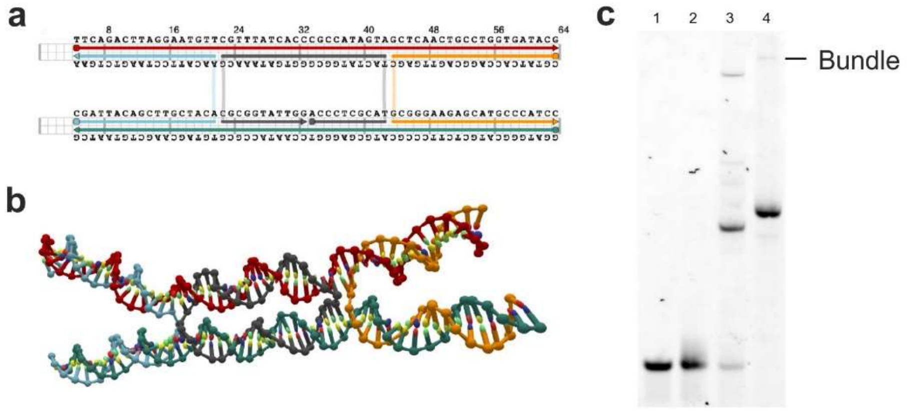

Figure S3. Design & assembly of the two-helix bundle. (**a**) scadnano[5] design of the Two-Helix Bundle. (**b**) OxDNA[6-8] representation of the designed Two-Helix Bundle (**c**) Formation gel: each lane contains the unpurified products of assembly of one more strand than the previous lane. Lane 1: S1; Lane 2: S1 + B_s2; Lane 3: S1 + B_s2 + S2; Lane 4: S1 + B_s2 + S2 + B_s1; Lane 5: S1 + B_s2 + S2 + B_s1 + B_s3

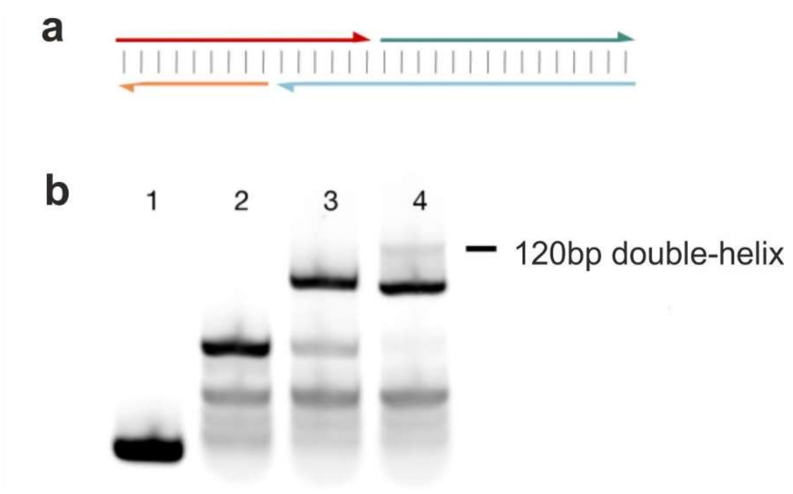

Figure S4. Design & assembly of the 120bp DNA helix. (**a**) 120 bp DNA Helix (**b**) Formation gel: each lane contains the unpurified products of assembly of one more strand than the previous lane. Lane 1: S1; Lane 2: S1 + Ds_s2; Lane 3: S1 + Ds_s2 + S2; Lane 4: S1 + Ds_s2 + S2+ Ds_s1.

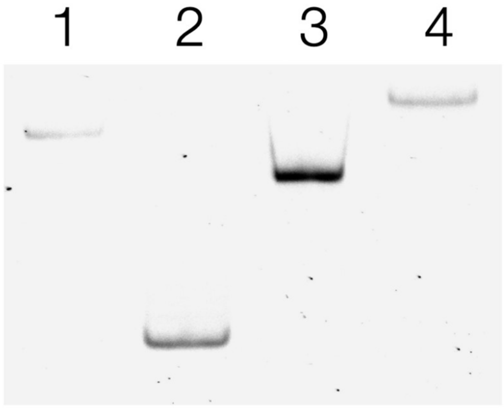

Figure S5. Native PAGE of all four gel purified structures imaged in the Atto532 fluorescence channel. Lane 1: Double-stranded DNA; Lane 2: Two-Helix Bundle; Lane 3: Square Tile; Lane 4: Tetrahedron.

Table S1. Tetrahedron strand sequences. All sequences are written 5' to 3'.

**T_s1**	AGGCAGTTGAGACGAACATTCCTAAGTCTGAAATTTATCACCCGCCATAGTAGACGTATCACC
**T_s2**	(5'ATTO532)CTTGCTACACGATTCAGACTTAGGAATGTTCGACATGCGAGGGTCCAATACCGACGATTACAG
**T_s3**	GGTGATAAAACGTGTAGCAAGCTGTAATCGACGGGAAGAGCATGCCCATCCACTACTATGGCG
**T_s4**	CCTCGCATGACTCAACTGCCTGGTGATACGAGGATGGGCATGCTCTTCCCGACGGTATTGGAC

Table S2. Square Tile strand sequences. All sequences are written 5' to 3'.

**Sq_s1**	(5'ATTO 532) TTCAGACTTAGGAATGTTCGTTTATCACCC
**Sq_s2**	GCCATAGTAGCTCAACTGCCTGGTGATACGGGATGGGCATGCTCTTCCCGCATGCGAGGG
**Sq_s3**	TCCAATACCGCGTGTAGCAAGCTGTAATCG
**Sq_s4**	CGTATCACCAGGCAGTTGAGGGGTGATAAACGAACATTCCTAAGTCTGAAATGCCCATCC
**Sq_s5**	TTGCTACACGCGGTATTGGACTACTATGGCCCCTCGCATGCGGGAAGAGCCGATTACAGC

Table S3. Two-Helix Bundle strand sequences. All sequences are written 5’ to 3’.

**S1**	(5’ATTO532)TTCAGACTTAGGAATGTTCGTTTATCACCCGCCATAGTAGCTCAACTGCCTGGTGATACG
**S2**	GGATGGGCATGCTCTTCCCGCATGCGAGGGTCCAATACCGCGTGTAGCAAGCTGTAATCG
**B_s1**	CGTATCACCAGGCAGTTGAGCGCGGGAAGAGCATGCCCATCC
**B_s2**	ACCCTCGCATTACTATGGCGGGTGATAAACGCGCGGTATTGG
**B_s3**	CGATTACAGCTTGCTACAAACATTCCTAAGTCTGAA

Table S4. 120bp DNA helix strand sequences. All sequences are written 5' to 3'.

**S1**	(5'ATTO532)TTCAGACTTAGGAATGTTCGTTTATCACCCGCCATAGTAGCTCAACTGCCTGGTGATACG
**S2**	GGATGGGCATGCTCTTCCCGCATGCGAGGGTCCAATACCGCGTGTAGCAAGCTGTAATCG
**Ds_s1**	GCATGCGGGAAGAGCATGCCCATCCCGTATCACCAGGCAGTTGAGCTACTATGGCGGGTGATAAACGAACATTCCTAAGTCGTAA
**Ds_s2**	CGATTACAGCTTGCTACACGCGGTATTGGACCCTC

Table S5. Physical properties of DNA nanostructures. Nanostructure properties listed in the table are as follows: the longest nanostructure dimension, l, calculated assuming 0.34 nm per base pair; total number of bases, n_{b}; number of individual single strands in the nanostructure, N_{s}; number of Atto532 fluorophores, N_{dye}; structural charge, q_{str}, calculated for each nanostructure using $-[n_{\mathrm{b}}$ +(N_{s}-1)+ $N_{\mathrm{dye}}]$, and for the calibrator molecule using $-[n_{\mathrm{b}}+ N_{\mathrm{dye}}]$.

Nanostructure	l (nm)	n_{b}	N_{s}	N_{dye}	q_{str} (e)
60ds rod (calibrator)	20.4	120	2	2	-122
120ds rod	40.8	240	4	1	-238
bundle	20.4	240	5	1	-237
tile	10.4	240	5	1	-237
tetrahedron	5.44	252	4	1	-250

2. Escape-time electrometry (ETe) to measure the effective charge, q_{m}, of DNA nanostructures

Overview of method

We use ETe to measure electrostatic interaction energies and therefore the effective electrical charge of molecules in solution, with high precision. Briefly, the method relies on optical observation of charged molecules diffusing in an electrostatic interaction energy landscape generated in a fluid-filled gap between parallel like-charged surfaces (Figure S6a). We measure the reduction in system free energy associated with transferring an electrically charged object from regions in which the parallel-plates are closely spaced into nanostructured ‘trap’ regions of very weak confinement. At the location of a trap, the two flanking surfaces are much further apart than in the slit region, and the molecule-plate electrical repulsion here is therefore negligible (Figure S6c). We create an array of such electrostatic fluidic traps using periodic nanoscale indentations in the upper plate of a slit created by two parallel silica surfaces, where the typical height of the gap is $2h = 70$ nm (Figure S6a).

Using wide-field fluorescence microscopy we observe the diffusive migration of molecules in a landscape of effectively identical electrostatic traps. We measure an average residence time, or escape time from the trap, t_{esc}, of the molecular species of interest. The escape time yields a sensitive measure of the depth of the potential well or the trap depth, W, created by the nanostructured surface indentations, which in turn depends strongly on the effective charge of the molecular species. In order to determine the effective charge we first infer a value of potential well depth, W, from our measurement of t_{esc}, and then determine the electrostatic contribution, ΔF_{el}, to the total trap depth, in order to infer a value of the measured effective charge, q_{m}.The experimentally inferred effective charges are denoted as q_{m} to distinguish a measured quantity from a theoretically calculated quantity, q_{eff} . Note that due to

spatial and rotational fluctuations of the molecule, we have $W > \Delta F_{\mathrm{el}}$ in general, described in detail in section 2.4.

2.1: Measuring the average species escape times, t_{esc}, from an electrostatic fluidic trap.

DNA nanostructures were stored at a concentration of approximately 5-15 nM in TE buffer (10 mM Tris (tris(hydroxymethyl)aminomethane), 1 mM EDTA (Ethylenediaminetetraacetic acid) and 5 mM $MgCl_2$ (except the tile conformation which was stored with no $MgCl_2$). Immediately prior to measurement DNA nanostructures were diluted to a final concentration of 5-10 pM in buffer solutions of 1 mM Tris, ≈0.005x TE and ≈5 μM $MgCl_2$, with the final ionic strength, I, tuned by adding NaCl. We parametrize experimental conditions using the dimensionless product, κh, of inverse electrostatic screening length, κ (inverse Debye length), and h, which denotes half the height of the slit. κ is obtained from electrical conductivity measurements of the measured aqueous suspension in the device, after each ETe measurement (HORIBA LAQUAtwin), as described previously[9]. The slit height, $2h$, is characterized using atomic force microscopy (Asylum Research); the device fabrication procedure is similar to that in previous work[10].

For a single measurement of a nanostructure species at a specific ionic strength, molecules are loaded into nanoslits housing several arrays of traps using a vacuum suction system. Once loaded, the vacuum is turned off initiating free diffusion of the DNA nanostructures across the trap landscape. Fluorescent signals from individual nanostructures were recorded using a standard wide-field fluorescence microscope, imaged using 5ms exposure times at frame rates from 5 to 50 Hz for up to 1000 frames on a sCMOS camera (Prime 95B, Photometrics). More molecules were then loaded into the trap arrays, and the procedure repeated about 20 times per measurement. From these videos of DNA nanostructure diffusion in the electrostatic free energy landscape, molecular residence times, Δt, in each trap were extracted using customized video analysis software. The average escape time, t_{esc}, for

each measurement was determined by fitting the probability density distribution, $P(\Delta t)$, with a function the form $P(\Delta t) = \frac{A}{t_{\mathrm{esc}}}\exp(-\frac{\Delta t}{t_{\mathrm{esc}}})$, as described in previous work [4,11,12]. Previous studies confirm that photobleaching does not affect the measurement of escape times in our experiment[9].

2.2: Brownian dynamics (BD) simulations of escape process to convert the measured escape times, t_{esc}, to well depth, W, in the free energy landscape.

We converted measurements of escape time to potential well depth values, W, as described previously[4,9,11,12]. As a general principle, when $W > 4\ k_{\mathrm{B}}T$, we expect an exponential dependence of t_{esc} on W of the form

$$t_{\mathrm{esc}} = t_{\mathrm{r}}\exp(W/k_{\mathrm{B}}T). \quad \text{(S1)}$$

Here, t_{r} is an effective molecular position relaxation time which is inversely related to the molecular diffusion coefficient, D, i.e., $t_{\mathrm{r}} \propto \frac{1}{D}$. While equation S1 holds for large well depths and provides qualitative insight into the underlying dynamics, it does not strictly hold for well depths $W < 4\ k_{\mathrm{B}}T$. In practice we do not know *a priori* the value of W for a given molecular species. We therefore perform Brownian Dynamics (BD) simulations of the escape process in order to accurately relate t_{esc} to W as described previously[9,12]. We simulate the thermal motion of a charged object in an electrostatic energy landscape, where the object is characterised by a friction coefficient given by $6\pi v\eta r_{\mathrm{H}}$, while moving at velocity, v, through a medium of dynamic viscosity, η. The hydrodynamic radius, r_{H}, of the molecular species is determined using two-focus Fluorescence Correlation Spectroscopy (2fFCS) to measure the molecular diffusion coefficient, which in turn may be expressed in terms of the radius of an equivalent sphere, r_{H} (see section 2.3). The electrostatic potential landscape in the fluidic nanostructure is calculated by numerically solving the Poisson-Boltzmann (PB) equation, for our device geometry, subject to the appropriate boundary conditions, using COMSOL

Multiphysics as described previously[9,12]. The electrostatic interaction energy of the particle at any point $\mathbf{r}$ in the landscape, referenced to a free energy at infinite slit height, is given by $F_{\mathrm{el}} = q_{\mathrm{eff}}\phi(\mathbf{r})$, where $\phi(\mathbf{r})$ is the electrical potential at $\mathbf{r}$. A periodic array of axially symmetric potential wells, constructed from the minimum free energy profile of a charged object traversing a single trap, is used for subsequent BD simulations (main text Figure 1c).

Simulated trajectories of particle motion in the landscape are used to generate image stacks similar to the experimental movies, as described previously[12]. Briefly, simulated movies are analysed in the same way as experimental movies in order to extract escape times (or average residence times of particles at trap locations) as a function of known input well depths, W. While the generation of simulated movies fosters high precision measurements (<1% on q_{m}), movie generation is computationally expensive and not necessary for measurements with lower accuracy requirements (uncertainty around 5%). Here, we work directly with the spatial coordinates corresponding to the trajectory of particle motion in the landscape as described in detail previously[9]. We apply spatial entry and exit criteria to the simulated particle trajectory in order to define a trapping event. We point out that the molecular entry and exit criteria required in the trajectory-based analysis are initially determined by a tuning procedure which aims to recover the same escape time in both the simulated trajectory-based analysis and in the simulated movies. Here we first generate simulated movies of the escape process using the simulated molecular trajectories and the same signal-to-noise and brightness characteristics as the experiment. We then analyse the simulated movies in the same way as the experimental recordings. The entry and exit criteria in the trajectory-based analysis are fine-tuned until we obtain excellent agreement between average escape times from the particle trajectory and the simulated movie analyses. Thereafter we use just the simulated trajectory data, in conjunction with the entry and exit criteria, in order to theoretically determine escape time values that correspond to known input well depths, W. By comparing experimentally measured escape

times with theoretically expected escape times determined for a range of well depths, $W,$ for a particle of known r_{H}, we determine the value of the unknown well depth in the experiment.

We see from equation S1 that the escape time depends exponentially on W and hence on the effective charge of the molecule, but only linearly on its size. Therefore our measured q_{m} values do not depend sensitively on accurate knowledge of molecular size, and a reasonable estimate of r_{H} will generally suffice. Note that as W always remains unknown at the outset, we do not *a priori* use equation S1 to convert t_{esc} to W. Once a set of simulations is performed for various value of W, we generally find that an exponential function of the form shown in equation S1 may be fit in the high well depth regime. For the DNA nanostructures in the study, we find $t_{\mathrm{r}} \approx 1.9$ ms, while for 60bp DNA, $t_{\mathrm{r}} \approx 1.4$ ms, under the experimental sampling conditions.

2.3: Measurement of hydrodynamic radii, r_{H}, of DNA nanostructures.

Dual-focus fluorescence correlation spectroscopy (2fFCS) was used to determine the effective hydrodynamic radii, r_{H}, of the DNA nanostructures. A homebuilt dual-focus confocal epi-fluorescence microscope was used to record fluorescence signals from labeled nanostructures freely diffusing in solution. Temporal correlation of the signals from the two foci yields a characteristic timescale for the diffusive transport of a molecular species between the two optical foci, from which the hydrodynamic radius can be inferred.

FCS measurements were performed on DNA nanostructures at 1-10 nM concentration in a buffer containing 10 mM Tris, 1 mM EDTA and 5 mM $MgCl_2$ (no $MgCl_2$ was used for measuring the tile sample). The molecules are excited by 5ps pulses of 520±15 nm laser light (NKT Photonics EXB-6 supercontinuum white light source, in conjunction with an NKT Photonics superK Varia wavelength selector) at 25 ns intervals alternatively at two confocal foci, spatially separated by a known distance. The pulses at 20 MHz are separated by a polarizing beam-splitter (Thorlabs), with a 25 ns temporal delay imposed on one arm via ~7.5

m of optical fiber. A Nomarski prism (Olympus) before the objective (1.2NA 60x Olympus) is then used to recombine the beams such that the two polarizations are brought to separate foci separated by a well-defined distance. The polarization selected time tagged time-resolved (TTTR) fluorescent signal is recorded on two avalanche photodiode single photon detectors (Micro Photon Devices, band-passed using filters from Chroma, Thorlabs and Semrock), and the cross-correlation function (CCF) between the TTTR signals is calculated (Figure S6a). Cross-correlation yields a timescale for diffusion between the optical foci, and hence a characteristic diffusion coefficient, D, for the species as outlined by Dertinger *et al.*[13]. The hydrodynamic radius, r_{H}, is calculated from D using the Stokes-Einstein equation $r_{\mathrm{H}} = k_{\mathrm{B}}T/6\pi\eta D$, with η the viscosity of water at temperature, T (Figure S6b).

We note that despite a significant disparity in the largest characteristic dimension of the nanostructures (compare a length of 41 nm for the cylindrical 120 bp DNA with ca. 8 nm for the side of the tetrahedron), and a corresponding variation of about a factor of 3 in the radius of gyration, R_{g}, the hydrodynamic radius has approximately the same value of $r_{\mathrm{H}} \approx 6 - 7$ nm for all DNA nanostructures (Figure S6b). We also point out that for all the DNA nanostructures in this study the electrostatic R_{g} turns out to be nearly identical to the standard definition of radius of gyration based on atomic positions in the molecule.

Measurement uncertainty in r_{H} is attributed to both measurement uncertainty in the diffusion coefficient, D_{e}, and in temperature, T_{e}, which influences r_{H} both explicitly through the Stokes-Einstein equation and also implicitly through its effect on viscosity, η. D_{e} is quantified by statistical bootstrapping which involves averaging over several fitting repetitions

of the CCF, each with a different random subset of all recorded data. T_e, is determined by the precision of the lab thermometer. Both uncertainties are accounted for in the quoted error, $r_{H,e}$.

2.4: Extraction of measured values of effective charge, q_m, from W

Having converted the measured escape times to potential well depth values, W, we next determine the electrostatic interaction free energies, ΔF_{el}, from which we deduce measured effective charge values, q_m, as described in previous work and summarized briefly here[4,9]. The well depth, W, can be broken down into the sum of two contributions: the free energy difference, ΔF_{el}, for states corresponding to electrostatic energy minima when the object is located at the midplane of the slit and the pocket regions, and a fluctuation contribution, f, that accounts for the difference in free energy due to axial and rotational motion of the object at each location[4,9,12,14]. ΔF_{el} is the difference in electrostatic free energy of interaction, $\Delta F_{el} = F_{el}|_{slit} - F_{el}|_{pocket} = q_{eff}(\phi_{mid}|_{slit} - \phi_{mid}|_{pocket})$, for a molecule positioned at the local energy minimum in the slit region, referenced to that in the pocket region[9,14]. In the absence of spatial fluctuations we would have $f = 0$ and therefore $W = \Delta F_{el}$ But since thermal fluctuations are always present, we write in general

$$W = W|_1 - W|_2 = \Delta F_{el} + f. \qquad \text{(S2)}$$

F_{el} has robust theoretical underpinnings in the Poisson-Boltzmann (PB) framework for solution phase electrostatics, and can be calculated by first solving the non-linear PB equation,

$$\nabla^2\psi = \kappa^2 \sinh\psi \qquad \text{(S3)}$$

subject to the appropriate boundary conditions for the experimental system as described previously[14,15] (see section 3 for further detail). Here $\psi = e\phi/k_BT$ is the dimensionless local electrical potential, ϕ. We have previously shown that ΔF_{el} is given by the product of an effective charge of the molecule in solution, q_{eff}, and the difference in midplane electrical potential, $\Delta\phi_{mid}$, between the slit and nanoscale pocket regions[14] (main text Figure 1).

Denoting the electrical potential at the midpoint between the parallel-plate slit surfaces as ϕ_{m}, and recognizing that $\phi_{\mathrm{mid}} = 0$ by design in the pocket region, we therefore have

$$\Delta F_{\mathrm{el}} = q_{\mathrm{eff}}\phi_{\mathrm{m}} \qquad \text{(S4)}$$

We emphasize that the relationship $F_{\mathrm{el}} = q_{\mathrm{eff}}\phi(\mathrm{r})$, on which Eq. S4 is based, has been shown to hold rigorously in theoretical treatments such as Kjellander's "dressed ion" theory[16,17].

Thus in order to obtain a measure of ΔF_{el} and hence of q_{eff} from the inferred well depth, *W*, we first need to determine the fluctuation contribution, *f*, to the total well depth, W. The dominant contribution to the fluctuation free energy comes from axial fluctuation of the molecule in the z-dimension in the slit as described previously[4,14]. There is also a small contribution from a rotational term which we consider for non-spherically symmetric objects. We determine $f = \Delta F_{\mathrm{trans}} + \Delta F_{\mathrm{rot}}$ by considering the differences in an 'excess' free energy due to translational ($\Delta F_{\mathrm{trans}}$) and rotational ($\Delta F_{\mathrm{rot}}$) fluctuations of the molecule between the two states of our parallel plate system. $\Delta F_{\mathrm{trans}}$ accounts for the thermal motion of a molecule about the minimum-energy midplane of the system. As shown previously, we can write $\Delta F_{\mathrm{trans}}$ in terms of the partition function, *Z*, of a point charge diffusing freely in *z* as follows[4]:

$$\Delta F_{\mathrm{trans}} = -k_{\mathrm{B}}T\ln Z|_1 + k_{\mathrm{B}}T\ln Z|_2 - q_{\mathrm{eff}}\phi_{\mathrm{m}} \qquad \text{(S5)}$$

$$Z = \int_0^{z_{\max}} e^{-\Delta F_{\mathrm{el}}(z)/k_B T}\, dz \qquad \text{(S6)}$$

where $\Delta F_{\mathrm{el}}(z) = q_{\mathrm{eff}}\phi(z)$ (S7)

represents the electrostatic free energy of the charged object located at any axial position, *z*, in the slit (referenced to the global minimum in interaction energy ($z \to \infty$)).

Solving the equation S3 for a monovalent, binary and symmetric electrolyte, subject to constant charge boundary conditions, shows that the electrical potential at a location *z* in a parallel plate slit can be approximated as $\phi(z) = \phi_{\mathrm{mid}}\cosh\left[\kappa\left(z - \frac{z_{\max}}{2}\right)\right]$.

Using equations S6 and S7, we evaluate *Z* for particle states 1 and 2 (the "slit" and "pocket" states, see Fig. S8a), with upper limits on the integral given by $z_{\max} = 2h$ or

$(2h + d)$, dictated by the maximum parallel plate separation in the slit $(2h)$ or in the pocket region $(2h + d)$. Note that $\phi(z_{\text{max}}/2) = \phi_{\text{mid}}$ is the electrical potential at the midplane of the slit.

ΔF_{rot}, in turn, accounts for the additional free energy due to rotational motion of a non-spherical particle. By approximating the DNA nanostructure as a line charge of length, l, given by the longest dimension of the nanostructure, carrying a charge density $\sigma_l = q_{\text{eff}}/l$, we can write ΔF_{rot} in terms of the partition function, Ω, of a freely rotating rod at an angle, θ, to the *z*-axis, as follows:

$$\Delta F_{\text{rot}} = -k_B T \ln\Omega|_1 + k_B T \ln\Omega|_2 - q_{\text{eff}}\phi_{\text{m}} \quad \text{(S8)}$$

Here we write the following expression for the local angular electrostatic energy, $\Delta F_{\text{rot}}(z,\theta)$, of a rod of length l located at a height *z* far away from the slit walls, oriented at an angle θ to the *z*-axis

$$\Delta F_{\text{rot}}(z,\theta) = \frac{1}{\cos\theta}\int_{z-\frac{l}{2}\cos\theta}^{z+\frac{l}{2}\cos\theta} \sigma_l \phi(z) dz \quad \text{(S9)}$$

We may also write the above equation in simpler form: $\Delta F_{\text{rot}}(z,\theta) = q_{\text{eff}}\langle\phi(z)\rangle$, where $\langle\phi(z)\rangle$ denotes the average electrical potential evaluated over a height in the slit ranging from $z - \frac{l}{2}\cos\theta$ to $z + \frac{l}{2}\cos\theta$. For example, for $\theta = 90°$, and $z = z_{\text{max}}/2$, equation S9 reduces to $\Delta F_{\text{rot}}(z,\theta) = q_{\text{eff}}\phi_{\text{mid}}$, which is the electrostatic interaction energy of a rod of charge q_{eff} lying along the mid-plane of the slit. Since the problem is azimuthally symmetric around the *z*-axis of the slit, the angular partition function for a rod centred on and confined to the midplane of the parallel-plate gap at $z = z_{\text{max}}/2$ is given by

$$\Omega = 4\pi \int_0^{\pi/2} e^{-\Delta F_{\text{rot}}(z_{\text{max}}/2,\theta)/k_B T} \sin\theta \, d\theta \quad \text{(S10)}$$

In order to simplify the calculation of the rotational term, we have assumed that the charged object is confined to the midplane of the system, i.e. we only consider rotational contributions for the molecule located at $z = z_{\text{max}}/2$. The accuracy of this approximation, which permits

greatly simplified calculation of ΔF_{rot}, was verified by comparison with an explicit full translational-rotational free energy calculation.

When calculating ΔF_{rot} for the different DNA nanostructures, we consider rods of different length, reflecting the longest dimension of the nanostructure. We assume a length of 0.34 nm per base pair which gives nanostructure lengths, l, as shown in Table S5. Since ΔF_{rot} depends on l, we find that $\Delta F_{\mathrm{rot}} \approx 0.8 k_{\mathrm{B}}T$ for the 120bp double helix, $\Delta F_{\mathrm{rot}} \approx 0.25 k_{\mathrm{B}}T$ for the bundle and 60bp helix and $\Delta F_{\mathrm{rot}} \approx 0.005 k_{\mathrm{B}}T$ for the tile and tetrahedron. This value may be compared with the $\Delta F_{\mathrm{trans}}$ contribution which is about $3 k_{\mathrm{B}}T$ in general, so that ΔF_{el} is about $1.65 k_{\mathrm{B}}T$ for the 120bp rod and $1 k_{\mathrm{B}}T$ for the tetrahedron under typical measurement conditions in this work. We emphasize that although the above analysis would appear to suggest requirement of knowledge of the unknown quantity q_{eff} at the outset, in practice this is not the case. This is because whereas ΔF_{el} depends linearly on q_{eff}, both $\Delta F_{\mathrm{trans}}$ and ΔF_{rot} depend weakly (logarithmically) on q_{eff}, and are therefore largely insensitive to q_{eff}[4]. Thus initial, approximate estimates of q_{eff} for each species suffice to determine $\Delta F_{\mathrm{trans}}$ and ΔF_{rot}, and the procedure may be repeated iteratively to refine the final estimate of measured effective charge q_{m}, if needed.

2.5: Calibration of the ET*e* measurement

We see from equation S4, that to make an accurate inference on q_{eff} from the inferred value of ΔF_{el} we also require accurate knowledge of ϕ_{m}, the electrical potential at the midplane of the slit. ϕ_{m} depends on the effective surface electrical potential at the silica walls of the slit, ϕ_{s} as follows

$$\phi_{\text{m}} = 2\phi_{\text{s}}\exp(-\kappa h) \tag{S11}$$

Treating ϕ_{s} as constant for experiments performed over a narrow range of salt concentrations (1.1-5.5 mM) in a given device, and combining equations S4 and S11 yields

$$\ln\frac{\Delta F_{\text{el}}}{k_{\text{B}}T} = \ln\frac{2q_{\text{eff}}\phi_{\text{s}}}{k_{\text{B}}T} - \kappa h \tag{S12}$$

We measured ΔF_{el} for all the molecular species in our study under various conditions of salt concentration and slit height and plotted the measurements vs. a dimensionless system size, κh (Figure S7b). Fits of the data to equation S12 yield values for the y-intercept given by $2q_{\text{eff}}\phi_{\text{s}}$ in each case. Knowing the value of the surface potential ϕ_{s} in the device would permit us to determine q_{eff} for each molecular species. Since an accurate value of ϕ_{s} is not available *a priori* we perform a calibration measurement using a well-characterized molecular species, whose geometry is well defined and whose effective charge, q_{eff}, is known from calculation[14]. Here, we typically use a 60bp double-stranded DNA fragment carrying two fluorophore labels as a calibration molecule, for which we expect $q_{\text{eff}} \cong -45\ e$ for measurements performed at ≈1 mM monovalent salt, similar to previous work[12,15]. Corresponding measured effective charge values, denoted as q_{m}, are displayed in Figure S7b. In general we find that the value of ϕ_{s} obtained for each device is stable over a timescale of several days and hence a single calibration run can be used for reliable measurements of effective charge, q_{m}, of further species under the same or similar conditions.

We point out that in order to enable a direct quantitative comparison of electrostatic interactions for the various molecules in the study, we work with measured effective charge values, q_{m}, rather than measured free energies, ΔF_{el}. This is because q_{m} is a property characteristic of the molecule and is robust to small variations in experimental conditions from one realization to the next. ΔF_{el} on the other hand, which is proportional to ϕ_{m}, is very sensitive to small variations in slit height and salt concentration, embodied in a single parameter κh as shown in equation S12 and discussed in Figure S7. Division of the measured

ΔF_{el} value by ϕ_{m} yields a measured value of effective charge, q_{m}, characteristic of the molecule of interest. Unlike t_{esc} and ΔF_{el}, q_{m} is robust to small changes in system size, κh, and permits direct comparison of measurements across independent realizations. Figure S7c displays individual measurements of ΔF_{el} converted to measured effective charge values, q_{m}, plotted vs. κh. Note that at $c = 1.5$ mM NaCl e.g., the slight dependence of η on salt concentration would imply $q_{\mathrm{eff}} \approx -46.2\ e$ for 60bp DNA, which is about 3% larger in magnitude than the value at 1 mM NaCl (outlined in section 3.3). However, η values for the DNA nanostructures are also expected to be similarly affected by salt concentration largely cancelling the slight influence of varying salt concentration in the final q_{eff} results (Figure S9). Measurements for all molecular species considered indeed show that q_{m} values remain constant within measurement uncertainty over a wider range of salt concentrations of ~1-6 mM (Fig. 1e).

2.6: Sources of error in q_{m}

In an ETe measurement that includes a calibration molecule, the uncertainty on t_{esc}, denoted as $t_{\mathrm{esc,e}}$, comes mainly from the statistical uncertainty of measuring *N* independent random events from a Poisson point process, and is given by $\frac{t_{\mathrm{esc}}}{\sqrt{N}}$ [11]. We point out that in an uncalibrated measurement a strong contribution to the measurement uncertainty stems from the uncertainty on κh, as described in detail previous work[4,12,14]. Use of a calibrator molecule in this work reduces the fractional measurement uncertainty and inaccuracy to a level essentially dictated by shot noise ($\frac{1}{\sqrt{N}}$).

As shown in previous work[12], the fractional error on q_{m}, may be estimated by

$$\frac{q_{\mathrm{m,e}}}{q_{\mathrm{m}}} = \frac{1}{\sqrt{N}} \frac{k_{\mathrm{B}}T}{\Delta F_{\mathrm{el}}} \quad \text{(S13)}$$

Since $\Delta F_{\mathrm{el}} \approx 1 - 2\ k_{\mathrm{B}}T$ in our measurements, we expect the fractional uncertainty on q_{m} to be comparable with the fractional statistical uncertainty on t_{esc}, which is simply $\frac{1}{\sqrt{N}}$. Measurements in this study are characterized by $N = 200 - 2000$ escape events, giving a measurement uncertainty of ≈2-7% on t_{esc} and on the molecular effective charge.

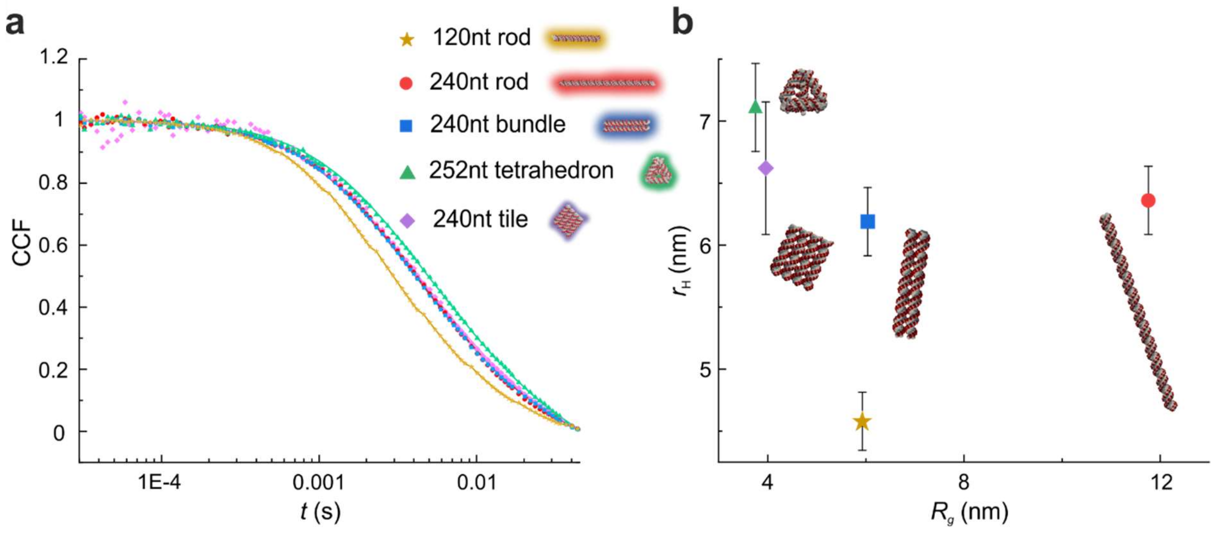

Figure S6. Measurement of hydrodynamic radii, r_{H}, by two-focus Fluorescence Correlation Spectroscopy (2fFCS). **(a)** The normalized cross-correlation function (CCF) was calculated for fluorescence emission recorded at separate detectors from labeled molecular species freely diffusing between two spatially separated, orthogonally polarized optical foci. A diffusion coefficient is extracted from fitting the CCF using the method outlined by Dertinger *et al.*[13], and the corresponding r_{H} value inferred from the Stokes-Einstein equation. (**b**) Measured r_{H} values for each nanostructure display little dependence on the molecular radius of gyration, R_{g}. Errors on r_{H} are evaluated as described in section 2.3.

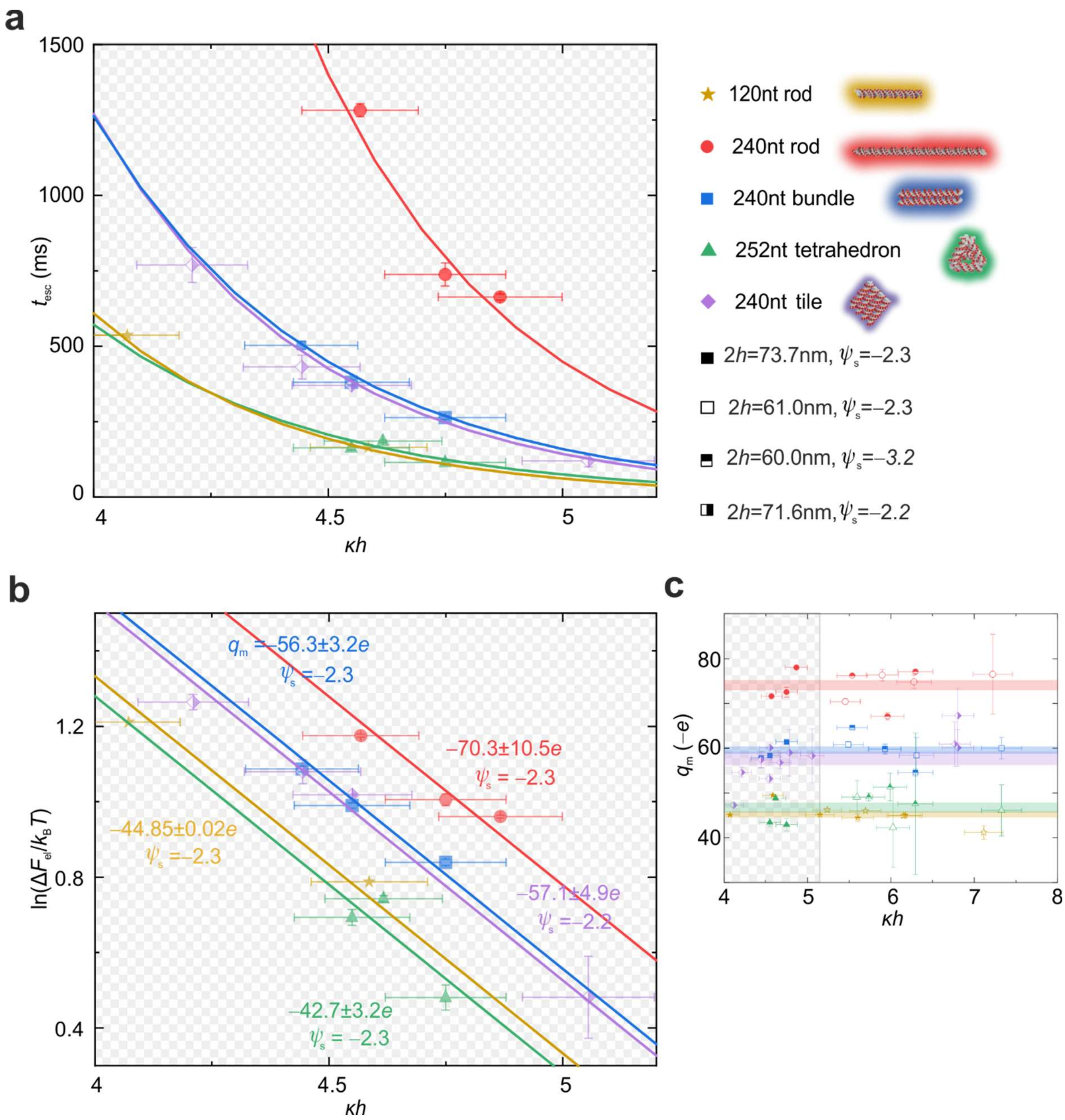

Figure S7. Measuring the effective charge of DNA nanostructures. (**a**) Escape times, t_{esc}, measured for the different DNA nanostructures in devices with slit depths $2h \approx$ 71.6 and 73.7 nm, and surface potentials $\psi_{\mathrm{s}} = -2.3$ to -2.2, over a range of Debye screening lengths, $\kappa^{-1} = 7 - 9$ nm, varied by changing the NaCl concentration, c. Error bars on t_{esc} values denote errors on fits of $P(\Delta t)$ vs. Δt data as shown in Figure 1 in the main text, while uncertainties in κh arise from accuracy limits on the measurement of slit height, h (uncertainty ~1 nm), discussed previously[9]. Lines are guides to eye. (**b**) Electrostatic trap depth (ΔF_{el}) inferred from the escape time measurements in (a), as described in the text. Fits of the data to equation S12 yield a measured molecular effective charge, q_{m}, with the silica surface potential, ψ_{s}, calibrated for each device using 60bp double-stranded DNA (as described in the text). (**c**) q_{m} values inferred for each individual measurement for all species are constant within measurement uncertainty for a given nanostructure species, over a range of salt concentrations ($c = 1.1 - 5.5$ mM) as well as across different measurement devices where slit heights ranged from $2h \approx 60.3$ to 73.7 nm and surface potentials ranged from $\psi_{\mathrm{s}} = -3.2$ to -2.2. Shaded region of the plot corresponds to the data range presented in (a) and (b). Data also shown in Figure 1e of the main text.

Procedure to calculate values of effective charge, q_{eff}, for DNA nanostructures

3.1: Free energy calculations to determine q_{eff}, for DNA nanostructures

As established in previous work[14,15] we determine the theoretical molecular effective charge, q_{eff}, by first calculating the electrostatic free energy difference, ΔF_{el}, associated with the trapping process. The electrostatic free energy, F, is determined from the dimensionless potential field, ψ, using the expression for the free energy functional

$$\frac{F}{k_{\mathrm{B}}T} = c\, N_{\mathrm{A}} \int_V [\kappa^{-2}|\nabla\psi|^2 - 2(\cosh\psi - \psi\sinh\psi - 1)]\, d\mathbf{x} \qquad \text{(S14)}$$

ψ is obtained by solving the non-linear Poisson-Boltzmann (NLPB) [equation S3] for the slit geometry (as shown in Figure 1a,c in the main text) using constant charge boundary conditions on all surfaces, including those of the slit and model DNA nanostructures, as described previously[14,15,18]. Here, the domain of interest is the electrolyte denoted by V, c is the bulk concentration of ionic species, and N_{A}, Avogadro's number. The integral in equation S14 is evaluated for the two trapping states: (1) Particle at the midplane of the slit region, oriented with the longest axis along the midplane ($\theta = 90°$), which represents the minimum electrical energy state of the object in the slit and (2) Particle at the minimum energy location in the "pocket" region. The difference between the two values yields the quantity that is measurable in the context of ET*e* experiments, i.e. $\Delta F_{\mathrm{el}} = F_1 - F_2$ (Figure S8). We have previously shown that ΔF_{el} is related to the effective charge via the midplane electrical potential in the slit, i.e., $q_{\mathrm{eff}} = \Delta F_{\mathrm{el}}/\phi_{\mathrm{m}}$[14].

Note that for calculations of the electrical free energy of a charged spheroid or the square-tile DNA nanostructure in bulk solution, as referred to in the analyses in Figures 3 and 4 of the main text, we evaluate equation S14 for a single state of the object immersed in an electrolyte. Here we solve the NLPB equation for the object immersed in a rectangular domain consisting only of bulk electrolyte, whose bounding surfaces are situated at a minimum

separation of $\approx 5\kappa^{-1}$ from the charge object, as shown in Figure S8. A zero electric field boundary condition on the domain surfaces ensures electroneutrality within the system.

3.2: Electrostatic modeling of DNA Nanostructures: molecular geometry and boundary conditions

We used COMSOL Multiphysics to solve equation S3 and evaluate equation S14 for DNA nanostructures in our device geometry. We constructed prototypes of origami structures from assemblies of cylinders, each representing a segment of double-helix in the nanostructure. We assume that the total structural charge, q_{str}, of the molecule is uniformly distributed over the surface area of the cylinders constituting the origami models, A_{m}; i.e. $\sigma_{\mathrm{m}} = q_{\mathrm{str}}/A_{\mathrm{m}}$. The radii of the cylinders, r_{cyl}, were assumed to be 12 Å, and the length of each cylinder, l, was determined by nb, where n is the number of base pairs in the particular segment of double-helix and $b = 3.4$ Å is the rise per basepair for a B-DNA double-helix. Thus a uniform, constant surface charge density, σ_{m}, was used as a boundary condition for the nanostructures. For the bundle and square-tile nanostructures, the nominal centre to centre separation between cylinders was assumed to be $s \approx 2.7$ Å[3]. However for the square-tile, we examined the influence of this parameter on the calculated value of ΔF_{el} by varying s over a range of approximately 2.6–4.5 Å (see main text Figure 4).

We neglect angular and spatial degrees of freedom in the calculation of ΔF_{el} (N.B. these degrees of freedom are removed from the experimental measurement prior to comparison with the theoretical value of ΔF_{el}, as described in section 2.4). We thus assume that the centre of mass of each DNA nanostructure lies in the midplane (local minimum of the electrostatic free energy) of a slit of height $2h = 75$ Å. For all planar nanostructure configurations (cylinder, bundle and square) we further assumed that the axes of all cylinders lie on the midplane of the slit (Figure S8b). However, in the special case of tetrahedron, the minimum energy configuration had one of the faces of tetrahedron parallel to the slit walls, with the centre of

geometry of the tetrahedron contained in the midplane of the slit (Figure S8c). Figure 2 of the main text presents these models together with electrical potential distributions both on the DNA nanostructure surfaces as well as in the electrolyte surrounding the model nanostructures in the pocket region. The boundary condition for the silica slit walls was a constant surface charge density, $\sigma_{\mathrm{w}} = -0.1e\ \mathrm{nm}^{-2}$, which is a typical value for silica interfaces in contact with aqueous electrolytes under our experimental conditions, as noted in previous work. We emphasize that the value of σ_{w} does not affect the results for q_{eff}.

A similar procedure was applied to the charged spheroids presented in the main text. For all spheroids, the centre of mass and major axis of the objects lie on the midplane of the slit. The aspect ratio of the spheroids was varied by changing the values of major and minor axes while constraining the volume of all objects to the volume of a sphere of radius 4 nm, corresponding to the radius of gyration of the DNA tetrahedron. A total structural charge of $q_{\mathrm{str}} = -240e$ was distributed uniformly over the surface of the spheroids, corresponding to a constant uniform charge density boundary condition on the particle surface.

3.3: Effect of salt concentration on effective charge s and electrostatic modelling of DNA Nanostructures

Figure S9 presents the impact of salt concentration on the calculated effective charge, q_{eff}, for the various DNA nanostructures. Taking $c = 1.5$ mM as a reference ionic strength, we normalize q_{eff} calculated at a salt concentration c for each DNA nanostructure to its corresponding value at $c = 1.5$ mM which we denote as q_0. A 30-50% change in salt concentration, c (corresponding to a change of ± 0.5 mM) alters the effective charge for 60bp double-stranded DNA by about $\pm 3\%$ which is of the same order or smaller than our measurement uncertainty on q_{m}. The renormalization factor, η, can be expected to vary substantially with salt concentration, e.g., by a factor of up to 4 over a change of 4 orders of magnitude in salt concentration for infinitely long rigid rods[14,19,20]. However, the range of

variation in η over our experimentally probed range of concentrations is of the order of 10% as shown in Fig. S9. Furthermore, we find a similar qualitative dependence of q_{eff} on salt concentration for all DNA nanostructures, which suggests that a large portion of the small salt concentration dependence of q_{eff} cancels out when the nanostructure measurements are referenced to that of the 60bp DNA calibrator molecule at each salt concentration

3.4: Comparison of renormalization factors calculated within our theoretical model with prior theoretical estimates

We have compared our calculated values of η to the results for infinite charged rods from previous theoretical work[21] and have found that our value of $\eta = 0.34$ for the 240nt case agrees quite well with the published value for infinite rods $\eta \approx 0.3$. Here the length of the rod is significantly longer than the Debye length and end effects could be expected to be less important. For the 240nt bundle, however the length of the effective cylinder is not substantially larger than the Debye length and end effects are expected to matter[21]. We find that for the bundle, our calculated of $\eta = 0.25$ agrees very well with our measurements, but is indeed slightly different from the value $\eta \approx 0.19$ suggested by Téllez & Trizac[22]. Here we use an assumption of equal surface areas in order to map the geometry of the bundle on to that of a simple cylinder. Finally, for the 120nt rod, our model of effective charge suggests $\eta = 0.38$, where the infinite rod model suggests $\eta \approx 0.3$. Overall, the comparison of our calculations for finite-length rods with a calculation for an infinite rod is not expected to be favourable for smaller rod lengths since end effects do begin to matter as the rod length approaches the Debye length.

We further note that the results for the square-tile nanostructure may be compared to those obtained for the idealized "thin disk". In order to enable such a comparison, we mapped the geometry of our square-tile on to that of a disc whose surface area corresponds to that of the square-tile and estimated an effective radius a for the square-tile. In the regime where $\kappa a <$

1, our calculated value of $\eta = 0.22$ can be compared with $\eta = 0.18$ obtained by Agra et al.[23]. Note that exact agreement is not expected given the approximations involved in making such a comparison.

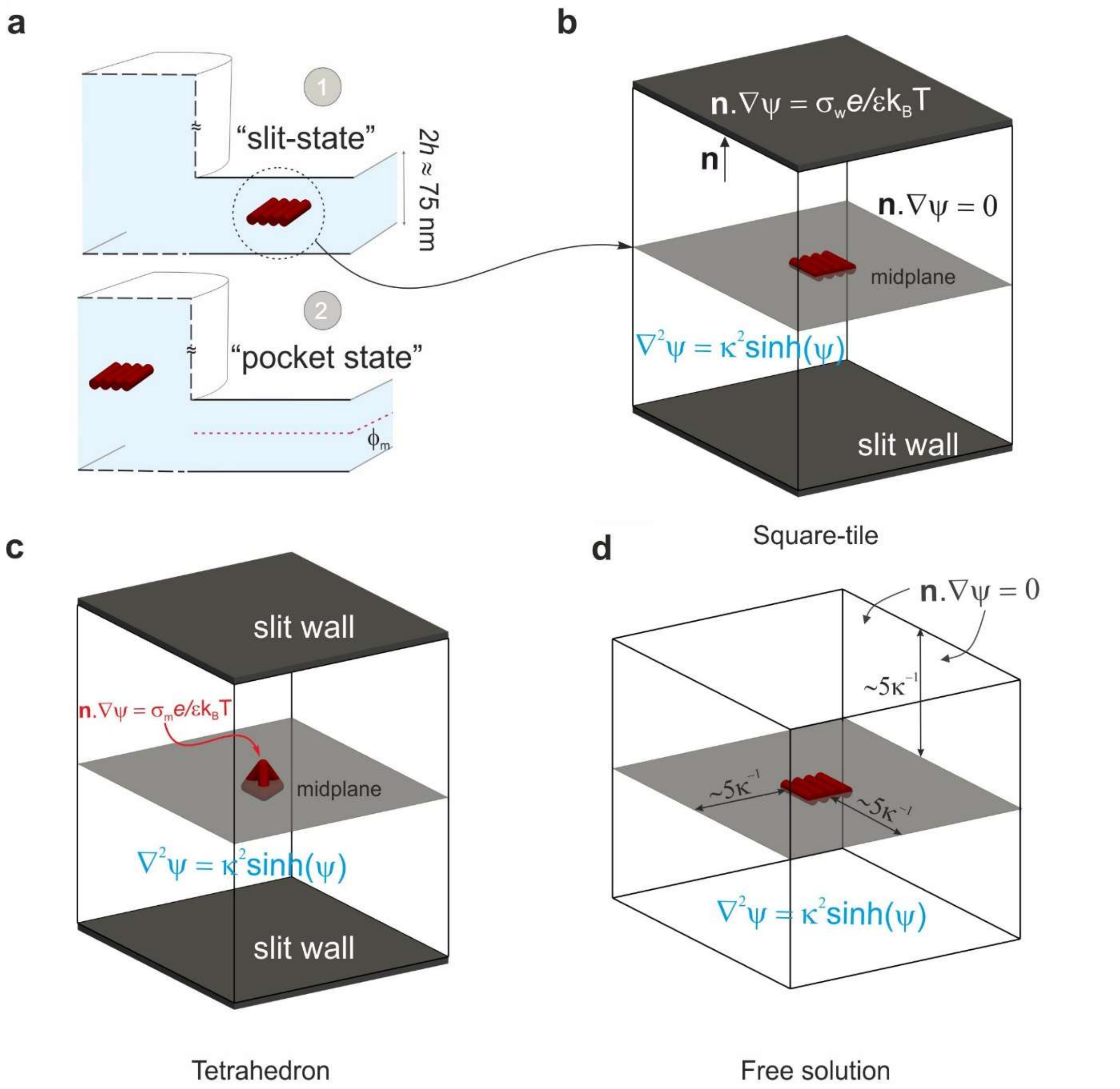

Figure S8. Schematic representations of DNA nanostructures in PB free energy calculations. (**a**) State (1) shows a square-tile origami in the slit region, and state (2) represents the trapped object in the pocket. (**b**) Position and orientation of a square-tile nanostructure in the slit state for free energy calculations. The governing PB equation in the electrolyte and the constant surface charge boundary conditions applied to the DNA nanostructures and slit surfaces are depicted. Note that n refers to the unit surface normal pointing out of the electrolyte. (**c**) Placement and orientation of a tetrahedron nanostructure in the slit for free energy calculations. (**d**) Geometry used for calculations of electrostatic free energy, *F*, in bulk solution for spheroids and square-tile structures shown in the main text Figures 3 and 4.

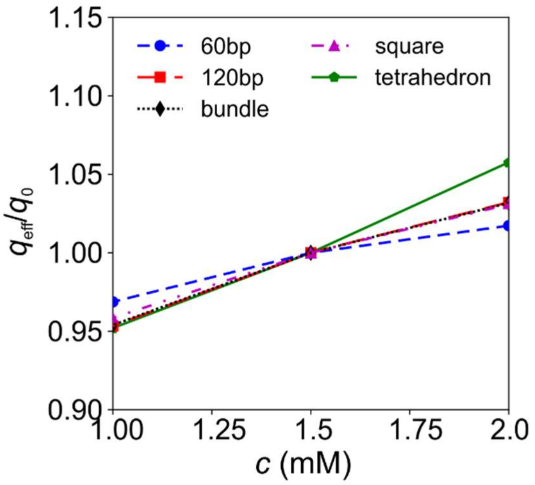

Figure S9. Impact of salt concentration on the effective charge, q_{eff}. Variation in salt concentration around a reference value ($c = 1.5$ mM) leads to small changes in the calculated effective charge. Graphs for the various DNA nanostructures display the ratio $\eta = q_{\mathrm{eff}}/q_0$ as a function of salt concentration, c, where q_0 is the effective charge of each nanostructure species at a reference salt concentration of $c = 1.5$ mM. $c = 1 - 2$ mM corresponds to $\kappa h = 3.7 - 5.2$, similar to the experiment shown in Figure S8a,b. For $c = 1.5$ mM we have $q_{\mathrm{eff}} = -46.2\ e$ for 60dsDNA.

REFERENCES

(1) Green, M. R.; Sambrook, J. Precipitation of DNA with ethanol. *Cold Spring Harbor Protocols* **2016**, *12*, 1116-1120.

(2) Goodman, R. P.; Schaap, I. A. T.; Tardin, C. F.; Erben, C. M.; Berry, R. M.; Schmidt, C. F.; Turberfield, A. J. Rapid chiral assembly of rigid DNA building blocks for molecular nanofabrication. *Science* **2005**, *310* (5754), 1661-1665.

(3) Fischer, S.; Hartl, C.; Frank, K.; Radler, J. O.; Liedl, T.; Nickel, B. Shape and interhelical spacing of DNA origami nanostructures studied by small-angle x-ray scattering. *Nano Lett.* **2016**, *16* (7), 4282-4287.

(4) Ruggeri, F.; Krishnan, M. Entropic trapping of a singly charged molecule in solution. *Nano Lett.* **2018**, *18* (6), 3773-3779.

(5) Doty, D.; Lee, B. L.; Stérin, T. scadnano: a browser-based, scriptable tool for designing DNA nanostructures. **2020**, 11841, *arXiv*. https://arxiv.org/abs/2005.11841 (accessed October 14, 2020).

(6) Ouldridge, T. E.; Louis, A. A.; Doye, J. P. K. Structural, mechanical, and thermodynamic properties of a coarse-grained DNA model. *J. Chem. Phys.* **2011**, *134* (8), 085101.

(7) Poppleton, E.; Bohlin, J.; Matthies, M.; Sharma, S.; Zhang, F.; Šulc, P. Design, optimization and analysis of large DNA and RNA nanostructures through interactive visualization, editing and molecular simulation. **2020**, *48* (12), e72.

(8) Poppleton, E.; Romero, R.; Mallya, A.; Rovigatti, L.; Sulc, P. OxDNA.org: a public webserver for coarse-grained simulations of DNA and RNA nanostructures. *Nucleic Acids Research* **2021**, *49* (W1), W491-W498.

(9) Ruggeri, F.; Zosel, F.; Mutter, N.; Różycka, M.; Wojtas, M.; Ożyhar, A.; Schuler, B.; Krishnan, M. Single-molecule electrometry. *Nature Nanotechnology* **2017**, *12* (5), 488-495.

(10) Krishnan, M.; Mojarad, N.; Kukura, P.; Sandoghdar, V. Geometry-induced electrostatic trapping of nanometric objects in a fluid. *Nature* **2010**, *467* (7316), 692-695.

(11) Ruggeri, F.; Krishnan, M. Spectrally resolved single-molecule electrometry. *J. Chem. Phys.* **2017**, *148* (12), 123307.

(12) Ruggeri, F.; Krishnan, M. Lattice diffusion of a single molecule in solution. *Physical Review E* **2017**, *96* (6), 062406.

(13) Dertinger, T.; Pacheco, V.; von der Hocht, I.; Hartmann, R.; Gregor, I.; Enderlein, J. Two-focus fluorescence correlation spectroscopy: a new tool for accurate and absolute diffusion measurements. *ChemPhysChem* **2007**, *8* (3), 433-443.

(14) Krishnan, M. A simple model for electrical charge in globular macromolecules and linear polyelectrolytes in solution. *J. Chem. Phys.* **2017**, *146* (20), 205101.

(15) Krishnan, M. Electrostatic free energy for a confined nanoscale object in a fluid. *J. Chem. Phys.* **2013**, *138* (11), 114906.

(16) Kjellander, R. & Mitchell, D. J. Dressed ion theory for electric double layer structure and interactions; an exact analysis. *Mol. Phys*. **1997**, *91* (2), 173-188

(17) Ulander, J., Greberg, H. & Kjellander, R. Primary and secondary effective charges for electrical double layer systems with asymmetric electrolytes. *J. Chem. Phys.* **2001**, *115* (15), 7144-7160.

(18) Theodoor, J.; Overbeek, G. The role of energy and entropy in the electrical double layer. *Colloids and Surfaces* **1990**, *51*, 61-75.

(19) Netz, R. R. & Orland, H. Variational charge renormalization in charged systems. *Eur. Phys. J. E Soft Matter* **2003**, *11* (3), 301-311.

(20) Aubouy, M., Trizac, E. & Bocquet, L. Effective charge versus bare charge: an analytical estimate for colloids in the infinite dilution limit. *J. Phys. A* **2003**, *36* (22), 5835-5840.

(21) Manning, G. S.; Mohanty, U. Counterion condensation on ionic oligomers. *Physica A* **1997**, *247* (1), 196-204.

(22) Téllez, G.; Trizac, E. Exact asymptotic expansions for the cylindrical Poisson–Boltzmann equation. *J. Stat. Mech.: Theory Exp.* **2006**, *2006* (06), P06018-P06018.

(23) Agra, R.; Trizac, E.; Bocquet, L. The interplay between screening properties and colloid anisotropy: Towards a reliable pair potential for disc-like charged particles. *Eur. Phys. J E* **2004**, *15* (4), 345-357.
